# Establishment of the Diagnostic Signature of Ferroptosis Genes in Multiple Sclerosis

**DOI:** 10.1007/s10528-024-10832-3

**Published:** 2024-06-17

**Authors:** Yang Yang, Qianqian Bai, Fangfei Liu, Shumin Zhang, Wenchao Tang, Ling Liu, Zhehua Xing, Hao Wang, Chi Zhang, Yanhui Yang, Hua Fan

**Affiliations:** 1 Office of Research & Innovation, The First Affiliated Hospital, College of Clinical Medicine, Henan University of Science and Technology, Luoyang, 471003 China; 2 Department of Trauma Center, The First Affiliated Hospital, College of Clinical Medicine, Henan University of Science and Technology, Luoyang, 471003 China

**Keywords:** Multiple sclerosis, Ferroptosis, Immune microenvironment, Genes, Diagnostic signature

## Abstract

Ferroptosis is a novel form of membrane-dependent cell death that differs from other cell death modalities such as necrosis, apoptosis, and autophagy. Multiple sclerosis (MS) is a chronic inflammatory disease of the central nervous system primarily affecting brain and spinal cord neurons. Although the pathogenesis of these two conditions may seem unrelated, recent studies have indicated a connection between ferroptosis and multiple sclerosis. In fact, ferroptosis plays a significant role in the development of MS, as evidenced by the presence of elevated iron levels and iron metabolism abnormalities in the brains, spinal cords, and other neurons of MS patients. These abnormalities disrupt iron homeostasis within cells, leading to the occurrence of ferroptosis. However, there is currently a lack of research on the diagnostic value of ferroptosis-related genes in multiple sclerosis. In this study, we employed bioinformatics methods to identify ferroptosis-related genes (*ATM, GSK3B, HMGCR, KLF2, MAPK1, NFE2L1, NRAS, PCBP1, PIK3CA, RPL8, VDAC3*) associated with the diagnosis of multiple sclerosis and constructed a diagnostic model. The results demonstrated that the diagnostic model accurately identified the patients’ condition. Subsequently, subgroup analysis was performed based on the expression levels of ferroptosis-related genes, dividing patients into high and low expression groups. The results showed differences in immune function and immune cell infiltration between the two groups. Our study not only confirms the correlation between ferroptosis and multiple sclerosis but also demonstrates the diagnostic value of ferroptosis-related genes in the disease. This provides guidance for clinical practice and direction for further mechanistic research.

**Supplementary Information:**

The online version contains supplementary material available at 10.1007/s10528-024-10832-3.

## Introduction

Multiple sclerosis (MS) is an autoimmune disorder that affects the central nervous system (CNS) (Axisa and Hafler 2016; Bar-Or and Li 2021). The primary pathological features of MS include extensive demyelination, infiltration of inflammatory cells, oligodendrocyte apoptosis, oxidative stress, and axonal injury (Dendrou et al. 2015; Kuhlmann et al. 2023a, b). The main clinical manifestations of MS are irreversible neurological deficits limb paralysis, vision loss, and cognitive impairments (Axisa and Hafler 2016; Kuhlmann et al. 2023a, b). Currently, the etiology of MS remains unclear. However, it appears to be influenced by both genetic susceptibility and environmental factors (Kuhlmann et al. 2023a, b; Rodríguez Murúa et al. 2022a). While MS is not solely a genetic disorder, certain genes do play a role in its onset and progression (Goris et al. 2022). Genome-wide association studies (GWAS) and human genome epidemiology (HuGE) studies have revealed that the primary signals of susceptibility for MS reside within the *HLA-DRB1* gene (Moutsianas et al. 2015; Hollenbach and Oksenberg 2015). The HLA-DRB1*15:01 allele had the most significant effect, exhibiting a notably elevated gene frequency compared to the control group, thereby elevating the risk of developing MS by approximately threefold (Beecham et al. 2013; Hafler et al. 2007). Additionally, numerous genes, such as *ZEB1*, have been demonstrated to play crucial roles in the pathogenesis and progression of MS via diverse mechanisms, including the activation of the JAK-STAT-signaling pathway, which facilitates abnormal Th1 and Th17 differentiation of CD4^+^ T cells (Qian et al. 2021). Therefore, conducting further research on the specific genes involved in the pathogenesis of MS play a crucial role in the diagnosis and treatment of MS.

Ferroptosis is a novel form of iron-associated cellular demise characterized by the excessive intracellular accumulation of free iron, resulting in oxidative stress that surpasses the cell’s antioxidant defense system and ultimately triggering lipid peroxidation, leading to cellular death (Dixon et al. 2012; Jiang et al. 2021; Tang et al. 2021). The mechanism underlying ferroptosis encompasses the intricate regulation of multiple biological processes, iron metabolism, membrane lipid metabolism, oxidative stress, and lipid radical generation (Sun et al. 2023; Liang et al. 2022). Recent investigations have unveiled a plethora of diseases associated with ferroptosis, encompassing trauma, cardiovascular and cerebrovascular disorders, tumors, as well as neurodegenerative conditions (Bersuker et al. 2019; Luoqian et al. 2022; Chen et al. 2021; Tang et al. 2021).

In recent years, the academic community has shown significant interest in exploring the pathological mechanism underlying MS and Ferroptosis (Hu et al. 2019; Sun et al. 2022; Luoqian et al. 2022). Research indicates a correlation between this mode of cell death and various nervous system diseases, including MS (Liang et al. 2022; Luoqian et al. 2022). Notably, lipid peroxidation of the cell membrane is a prominent characteristic of MS. Further investigations have revealed distinct features in cells and mitochondria within the central nervous system that render them susceptible to Ferroptosis, such as glucose transporter *GLUT1* and iron storage protein ferritin (Jiang et al. 2017; Hou et al. 2016). Furthermore, recent studies have identified abnormally elevated levels of iron in various tissues among patients diagnosed with MS (Costa et al. 2023; Luoqian et al. 2022). This phenomenon leads to heightened oxidative damage to cellular structures, particularly mitochondria, and triggers dysregulation of iron homeostasis, ultimately culminating in cellular apoptosis (Costa et al. 2023; Luoqian et al. 2022). Consequently, this intricate process exacerbates the progression and deterioration of the disease (Luoqian et al. 2022; Díaz et al. 2019). By comprehending the impact and molecular mechanism underlying ferroptosis, it becomes feasible to explore novel therapeutic strategies for alleviating symptoms and progression of MS, thereby offering an innovative approach for advanced-stage management of this disease. However, to date, no research has been conducted to investigate the diagnostic value of iron death-related genes in MS. In this study, we used bioinformatics to predict the role of iron death-related genes in multiple sclerosis and established a diagnostic prediction model, providing guidance for clinical and mechanistic research.

## Materials and Methods

### Data Downloading

We downloaded three expression profile datasets, GSE17048 (control = 45, multiple sclerosis = 99, all included in the study), GSE41848 (control = 79, multiple sclerosis = 133, all included in the study), and GSE21942 (control = 15, multiple sclerosis = 12, all included in the study), for multiple sclerosis (MS) from the GEO database (Barrett et al. 2007) using the R package GEOquery (Davis and Meltzer 2007). GSE17048 (Gandhi et al. 2010) and GSE41848 (Nickles et al. 2013) were used as the training sets while GSE21942 (Kemppinen et al. 2011) was used as the validation set. All three datasets were derived from humans (Homo sapiens), while GSE17048 and GSE41848 were obtained from whole blood and GSE21942 was obtained from peripheral blood mononuclear cells. In addition, in the three datasets, the sequencing platforms for GSE17048 were GPL6947 Illumina HumanHT-12 V3.0 expression beadchip, for GSE41848 were GPL16209 Affymetrix Human Exon 1.0 ST Array, and for GSE21942 were GPL570 Affymetrix Human Genome U133 Plus 2.0 Array. Detailed information can be found in Table 1. We downloaded the platform annotation information for each dataset to convert probe names to gene names, and replaced multiple expression results of specific genes with the mean value of expression levels. To obtain Ferroptosis-related genes (FRGs), we downloaded a Ferroptosis-related gene set (WP FERROPTOSIS) containing 64 FRGs from the Molecular Signatures Database (MSigDB) (Liberzon et al. 2015) using the keyword “Ferroptosis”. We also downloaded three Ferroptosis-related gene sets (Ferroptosis driver gene, Ferroptosis suppressor gene, Ferroptosis marker gene) from the Ferroptosis database (Zhou et al. 2023) and obtained 361 FRGs after merging and removing duplicates. Additionally, we searched the GeneCards database (Stelzer et al. 2016) using “Ferroptosis” as a keyword and retained only “Protein Coding” FRGs with a Score > 1, resulting in 265 FRGs. Finally, we obtained a total of 420 FRGs by taking the union and removing duplicates of the genes obtained from MSigDB, the Ferroptosis database, and GeneCards. Detailed information is provided in Table S1.

**Table 1:** GEO Dataset Information list

	GSE17048	GSE41848	GSE21942
Platform	GPL6947	GPL16209	GPL570
Species	Homo sapiens	Homo sapiens	Homo sapiens
Tissue	Whole blood	Whole blood	Peripheral blood mononuclear cells
Samples in Normal group	45	79	15
Samples in MS group	99	133	12

*GEO* Gene Expression Omnibus, *MS* Multiple sclerosis

### Differential Analysis

To obtain differentially expressed genes (DEGs) between the multiple sclerosis (MS) and normal control groups, we first used the R package sva (Leek et al. 2012; Ringnér 2008) to remove batch effects from datasets GSE17048 and GSE41848, resulting in a merged dataset containing 232 MS samples and 124 normal control samples. To validate the batch removal, we performed principal component analysis (PCA) (Ringnér 2008) on the dataset before and after batch correction. PCA is a method for data dimensionality reduction, extracting feature vectors (components) from high-dimensional data, transforming them into low-dimensional data, and using two-dimensional or three-dimensional plots to display these features. Next, we used the R package limma (Ritchie et al. 2015) to perform differential analysis based on the grouping information in the data, obtaining DEGs. Finally, we obtained the intersection of all DEGs with |logFC|> 0 and p.adjust < 0.05 from the merged dataset differential analysis and FRGs, and created a Venn diagram, then obtained ferroptosis-related differentially expressed genes (FRDEGs) by intersecting the resulting genes with DEGs obtained from the validation dataset GSE21942. We used the R packages ggplot to plot the volcano plot and pheatmap to visualize the heatmap of FRDEGs. In addition, we also plotted the grouping comparison graph and chromosome location map of FRDEGs under MS/Normal grouping.

### Correlation Analysis of FRDEGs

To explore the expression correlation of ferroptosis-related differentially expressed genes (FRDEGs) in the merged dataset, we used the ggplot package to draw correlation heatmaps and scatterplots of the FRDEGs in the merged dataset, and used the circlize package to draw correlation chord diagrams.

### Lasso-Logistic Regression Analysis

To investigate the differences in FRDEGs between the MS group and the Normal group, we performed LASSO (Least Absolute Shrinkage and Selection Operator) (Cai and van der Laan 2020) regression analysis using the glmnet package based on FRDEGs with parameter seed = 2022 and family = “binomial”. We ran 1000 cycles to prevent over fitting. LASSO regression is commonly used to construct prognostic models. Based on linear regression, it reduces overfitting and improves the generalization ability of the model by adding a penalty term (lambda x absolute value of the slope). Based on the results of LASSO selection, we performed single and multiple factor logistic regression analysis on the selected FRDEGs based on expression levels and generated a forest plot. We then plotted a calibration curve for the multiple factor logistic model. The calibration curve is a fit of the actual probability and the model-predicted probability under different circumstances. We also plotted a DCA (Decision Curve Analysis) curve and a ROC (Receiver Operating Characteristic) curve to evaluate the diagnostic accuracy of the logistic model.

### Molecular Subtype Analysis

Consensus clustering could differentiate samples into several subtypes based on different omics datasets, in order to discover new disease subtypes or perform comparative analysis among different subtypes. To explore possible disease subtypes that might be related to the expression of FRDEGs in the merged datasets of MS samples, we conducted clustering analysis using the R package ConsensusClusterPlus based on the expression of FRDEGs. The parameter settings were as follows: 100 repetitions (reps = 100), 80% resampling of samples (pItem = 0.8), 100% resampling of features (pFeature = 1), and PAM as the clustering algorithm. Based on the calculated results, we generated a heatmap of the clustering results, a cumulative distribution function (CDF) plot, and a Delta Area plot, as well as a grouped comparison of FRDEGs in different disease subtypes and ROC validation.

### Interaction Network of FRDEGs

Protein–protein interaction network (PPI Network) is composed of individual proteins interacting with each other. STRING database is a database that searches for known and predicted interactions between proteins. In this study, we used the STRING database, set the biological species to human, and used a minimum interaction score greater than 0.400 to construct a protein–protein interaction network for FRDEGs. The network was visualized using Cytoscape 38. In addition, we used the MCC (Maximal Clique Centrality) algorithm in the cytoHubba plugin to calculate and display the scores. ENCORI database (Li et al. 2014) is the 3.0 version of the starBase database. The interactions between miRNA-ncRNA, miRNA-mRNA, ncRNA-RNA, RNA-RNA, RBP-ncRNA, and RBP-mRNA in the ENCORI database were mined based on CLIP-seq and degradation sequencing (for plants) data, providing various visualization interfaces for exploring miRNA targets. miRDB database is used for miRNA target gene prediction and functional annotation. We used the ENCORI and miRDB databases to predict miRNA interactions with FRDEGs. Then, we drew an mRNA-miRNA interaction network by intersecting the miRNA-mRNA data with Target Score > 90 in the miRDB database and the mRNA-miRNA data in the ENCORI database. CHIPBase database (version 2.0) (https://rna.sysu.edu.cn/chipbase/) identifies thousands of binding motif matrices and their binding sites from ChIP-seq data of DNA-binding proteins and predicts the transcriptional regulatory relationships between millions of transcription factors (TF) and genes. hTFtarget database (http://bioinfo.life.hust.edu.cn/hTFtarget.) is a comprehensive database containing data on human transcription factors (TF) and their corresponding regulatory targets. We searched for TFs that interact with FRDEGs using the CHIPBase database (version 2.0) and hTFtarget database and visualized the mRNA-TF interaction network using Cytoscape software.

### Functional (GO) and Pathway Enrichment (KEGG) Analysis of FRDEGs

Gene Ontology (GO) enrichment analysis is a commonly used method for large-scale functional enrichment research on genes at different dimensions and levels, generally carried out from three levels: biological process (BP), molecular function (MF), and cellular component (CC). Kyoto Encyclopedia of Genes and Genomes (KEGG) is a widely used database that stores information on genomes, biological pathways, diseases, drugs, and more. We used R package clusterProfiler for GO functional annotation analysis and enrichment analysis of FRDEGs. The selection criteria for entries were *p* value < 0.05 and FDR value (*q* value) < 0.20, and the P value correction method was Benjamini–Hochberg method (BH).

### Gene Set Enrichment Analysis (GSEA)

GSEA is a computational method proposed by the Broad Institute to determine if a predefined set of genes shows statistical differences between two biological states, commonly used to estimate changes in pathway and biological process activity in expression data sets. To investigate biological process differences between two groups of samples, we downloaded the reference gene set “c2.cp.v7.2.symbols.gmt” from the MSigDB database based on gene expression profile data sets, and conducted enrichment analysis and visualization using the GSEA method included in the R package “clusterProfiler”. The parameters used in this GSEA are as follows: seed is 2020, calculation is performed 1000 times, each gene set contains at least 10 genes and at most 500 genes, the *p*-value correction method is Benjamini-Hochberg (BH), and the screening criteria for significant enrichment is *p*.adjust < 0.05 and FDR value (*q*.value) < 0.2.

### Immune Infiltration Analysis (CIBERSORTx)

CIBERSORTx is a R/web-based tool that deconvolutes the expression matrix of human immune cell subtypes based on linear support vector regression. It could evaluate the infiltration status of immune cells in sequencing samples based on the gene expression characteristic set of 22 known immune cell subtypes. In this study, the CIBERSORTx algorithm was used to evaluate the immune cell infiltration status of the merged dataset, and Spearman correlation was calculated to assess the relationship between different immune cells. Subsequently, we used the R package “ggplot2” to draw heat maps and correlation scatter plots for visualization.

### Construction of Immunophenotypic Subtypes

Single Sample Gene Set Enrichment Analysis (ssGSEA) could estimate the quantity of specific immune infiltrating cells and the activity of specific immune responses. The ssGSEA method employs an enrichment score to quantify the absolute enrichment of the gene set within each sample in the dataset. The marker genes for the 28 immune cell types were derived from a previously published article, a comprehensive set of 28 commonly observed immune cell populations and comprising a total of 782 genes (Subramanian et al. 2005). We used the ssGSEA algorithm in the R package GSVA to analyze the enrichment scores calculated for each sample to represent the infiltration level of each immune cell type in each sample. The correlation between immune infiltrating cells was determined by Spearman correlation analysis. Consensus clustering could differentiate samples into several subtypes based on different omics datasets to discover new disease subtypes or compare different subtypes. We used the R package ConsensusClusterPlus for clustering analysis, dividing disease group samples into different groups according to the infiltration level of immune cells based on the results obtained from ssGSEA to construct immunophenotypic subtypes. The parameters were set to repeat 100 times (reps = 100), resample the samples by 80% (pItem = 0.8), resample the features by 100% (pFeature = 1), and cluster using the PAM algorithm. Subsequently, we used the R package pheatmap to draw correlation heatmaps showing the correlation analysis results between FRDEGs and immune cells in the two immune subtypes of multiple sclerosis (MS).

### ROC Curve

Receiver operating characteristic (ROC) curve is a graphical tool that can be used to select the best model, discard suboptimal models, or set an optimal threshold in the same model. ROC curve is a comprehensive indicator reflecting the sensitivity and specificity of continuous variables, and represents the relationship between sensitivity and specificity through the construction of graph. The area under the ROC curve (AUC) is generally between 0.5 and 1, and the closer AUC is to 1, the better the diagnostic effect is. In general, AUC has low accuracy at 0.5–0.7, moderate accuracy at 0.7–0.9, and high accuracy at above 0.9. We used the pROC package to draw the ROC curve of FRDEGs in different groups (Normal/MS), calculate the AUC, and evaluate the diagnostic effect of FRDEG expression on the disease.

### Statistical Analysis

All data processing and analysis in this article are based on R software (version 4.2.1), and the presentation of continuous variables is shown as mean ± standard deviation. Wilcoxon Rank Sum Test is used to compare two groups, and chi-square test or Fisher’s exact test is used to compare and analyze the statistical significance between two groups of categorical variables. If not specifically stated, all results are calculated using Spearman correlation analysis to assess the correlation coefficient between different molecules, and all results are based on *p* < 0.05 as the standard for significant differences.

## Results

### Differential Expression of Ferroptosis-Related Genes in Patients with Multiple Sclerosis

The technical workflow of this bioinformatics analysis is shown in the Figure S1. First, we used the R package sva to remove batch effects from the datasets GSE17048 and GSE41848, and obtained a merged GEO dataset. We compared the datasets before and after batch effect removal using a density box plot and a Principal Component Analysis (PCA) plot (Figure S2A–D). The results of the density box plot and PCA plot showed that batch effects of the samples in the dataset were basically eliminated after batch effect removal.

**Fig. 1:**
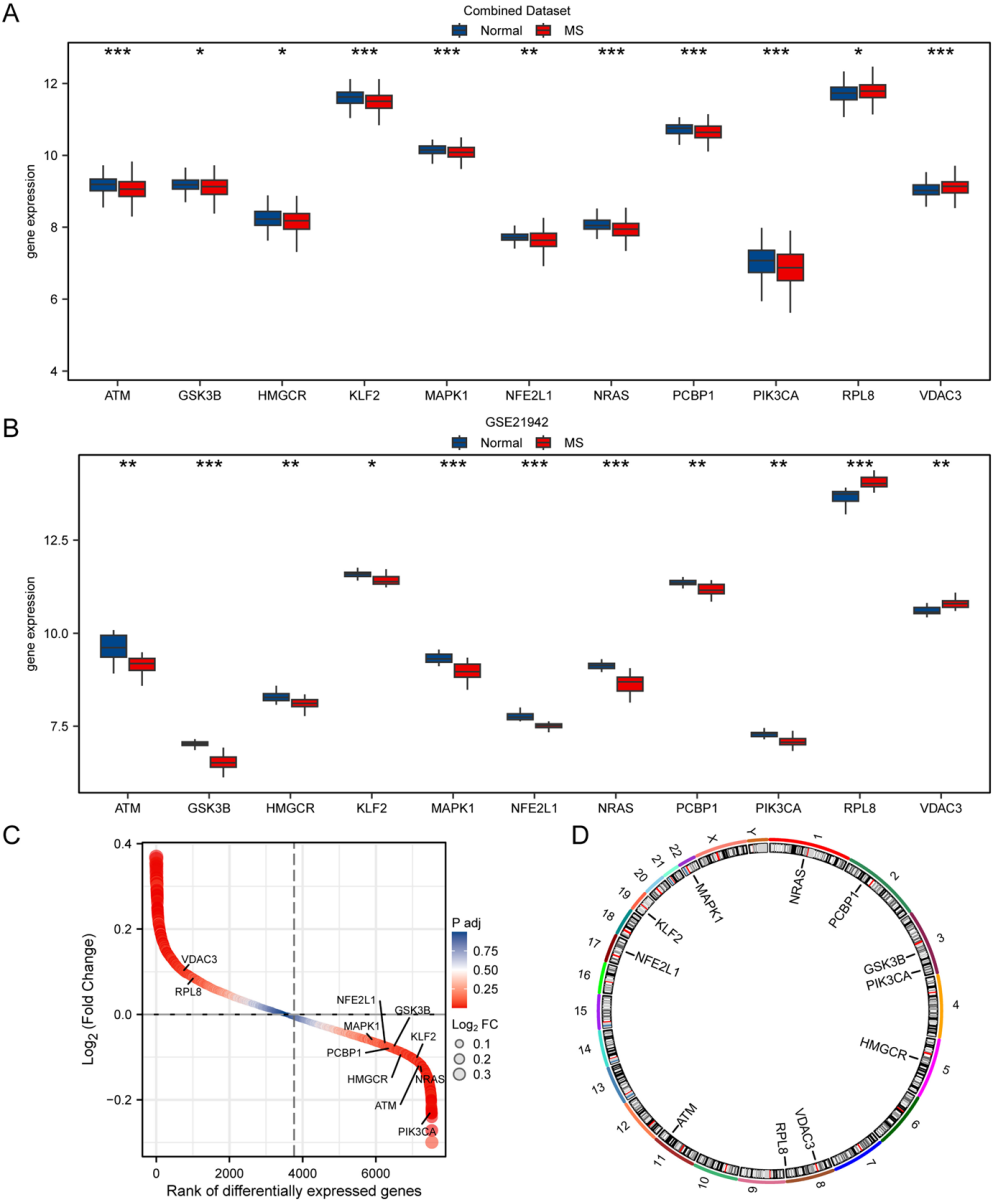
Expression difference analysis and chromosomal location of FRDEGs. **A** Expression difference analysis and chromosomal location of FRDEGs. **B** Group comparison plot of FRDEGs in MS and Normal groups in the merged dataset. **C** Group comparison plot of FRDEGs in MS and Normal groups in validation set GSE21942. **D** Differential ranking plot of FRDEGs in the merged dataset. Chromosomal location plot of FRDEGs. Symbol * is equivalent to P < 0.05, indicating statistical significance; symbol ** is equivalent to P < 0.01, indicating high statistical significance; symbol *** is equivalent to P < 0.001, indicating extremely high statistical significance

To analyze the differential gene expression between the control (Normal) and multiple sclerosis (MS) groups in the merged dataset, we used the R package limma to perform differential analysis and obtained the differentially expressed genes (DEGs) with thresholds of |logFC|> 0 and *p*.adjust < 0.05. In total, 1726 genes in the merged dataset were identified as DEGs, with 851 genes upregulated (logFC > 0 and *p*.adjust < 0.05) and 875 genes downregulated (logFC < 0 and *p*.adjust < 0.05) (Figure S3B, C). We intersected all DEGs with logFC > 0 and *p*.adjust < 0.05 and ferroptosis-related genes, resulting in 23 upregulated ferroptosis-related genes, and intersected all DEGs with logFC < 0 and *p*.adjust < 0.05 and ferroptosis-related genes, resulting in 36 downregulated ferroptosis-related genes (Figure S3E, F). Then, we intersected these intersected genes with the up/downregulated genes obtained from the validation dataset GSE21942 and identified 11 ferroptosis-related differentially expressed genes (FRDEGs) that were consistent in trend between the merged and validation datasets (*ATM, GSK3B, HMGCR, KLF2, MAPK1, NFE2L1, NRAS, PCBP1, PIK3CA, RPL8, VDAC3*), with 2 upregulated (*RPL8, VDAC3*) and 9 downregulated genes (*ATM, GSK3B, HMGCR, KLF2, MAPK1, NFE2L1, NRAS, PCBP1, PIK3CA*). The intersection relationships were presented in a Venn diagram (Figure S3A). DEGs of the MS and Normal groups in the validation set GSE21942 are shown as Venn diagram (Fig. 3D). Subsequently, based on the intersection results, we plotted the grouping comparison (Fig. 1A, B) and differential ranking (Fig. 1C) of the 11 FRDEGs in the merged dataset and validation set GSE21942. From Fig. 1A, B, it can be observed that all 11 FRDEGs exhibit significant differences between the MS group and Normal group in both the merged dataset and the validation set GSE21942. In addition, we also plotted the chromosome location map of FRDEGs (Fig. 1D), which shows that FRDEGs are mainly concentrated on chromosomes 1, 2, 3, 5, 8, 11, 17, 19, and 22.

### The Expression of FRDEGs Exhibits an Interactive Relationship

**Fig. 2:**
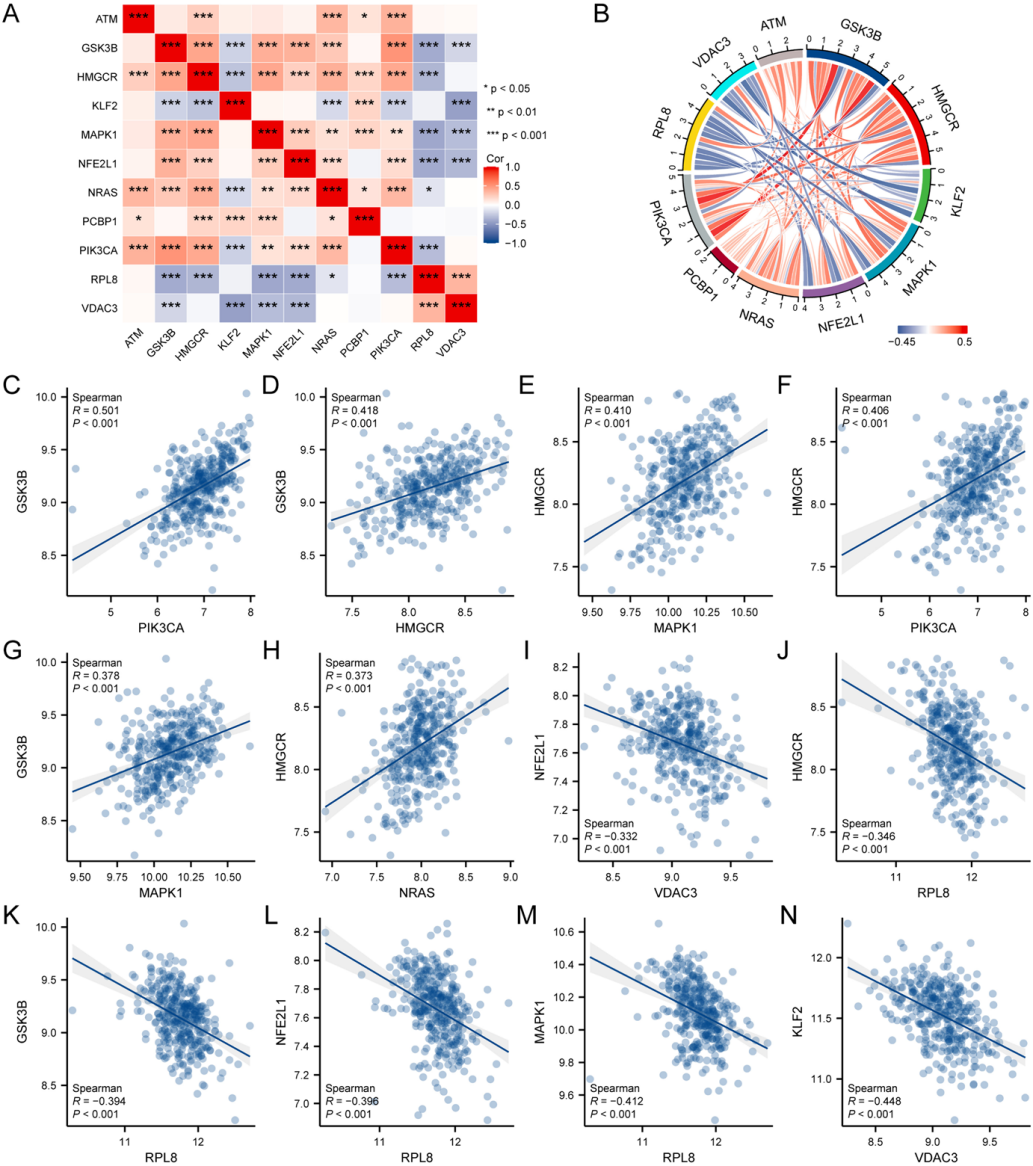
Correlation analysis of FRDEGs. **A** Heat map of the correlation of FRDEGs. **B** Chord diagram of the correlation of FRDEGs. **C**–**N** Scatter plots showing the correlation between *PIK3CA* and *GSK3B* (**C**), *HMGCR* and *GSK3B* (**D**), *MAPK1* and *HMGCR* (**E**), *PIK3CA* and *HMGCR* (**F**), *MAPK1* and *GSK3B* (**G**), *NRAS* and *HMGCR* (**H**), *VDAC3* and *NFE2L1* (**I**), *RPL8* and *HMGCR* (**J**), *RPL8* and *GSK3B* (**K**), *RPL8* and *NFE2L1* (**L**), *RPL8* and *MAPK1* (**M**), and *VDAC3* and *KLF2* (**N**). Symbols * represent P < 0.05, showing statistical significance. Symbols ** represent P < 0.01, indicating high statistical significance. Symbols *** represent P < 0.001, representing extremely high statistical significance. A positive correlation coefficient (r) indicates a possible positive correlation between two variables, whereas a negative correlation coefficient suggests a potential negative correlation between two variables. A correlation coefficient with an absolute value above 0.8 indicates a strong correlation; an absolute value between 0.5–0.8 represents a moderately strong correlation; an absolute value between 0.3–0.5 indicates a moderate correlation, and an absolute value below 0.3 represents a weak or no correlation

To explore the correlation of FRDEGs (*ATM, GSK3B, HMGCR, KLF2, MAPK1, NFE2L1, NRAS, PCBP1, PIK3CA, RPL8, VDAC3*) in the merged dataset, we plotted a heatmap (Fig. 2A) and a correlation chord diagram (Fig. 2B). We plotted the scatterplots for the top 12 correlated pairs of FRDEGs (Fig. 2C–N). The results showed that there was a moderate positive correlation between *PIK3CA* and *GSK3B* (Fig. 2C, r = 0.501); a general positive correlation between *HMGCR* and *GSK3B* (Fig. 2D, r = 0.418), *MAPK1* and *HMGCR* (Fig. 2E, r = 0.410), *PIK3CA* and *HMGCR* (Fig. 2F, r = 0.406), *MAPK1* and *GSK3B* (Fig. 2G, r = 0.378), and *NRAS* and *HMGCR* (Fig. 2H, r = 0.373); and a general negative correlation between *VDAC3* and *NFE2L1* (Fig. 2I, r = − 0.332), *RPL8* and *HMGCR* (Fig. 2J, r = − 0.346), *RPL8* and *GSK3B* (Fig. 2K, r = − 0.394), *RPL8* and *NFE2L1* (Fig. 2L, r = − 0.396), *RPL8* and *MAPK1* (Fig. 2M, r = − 0.412), and *VDAC3* and *KLF2* (Fig. 2N, r = − 0.448).

### FRDEGs had a Certain Degree of Diagnostic Accuracy for MS

**Fig. 3:**
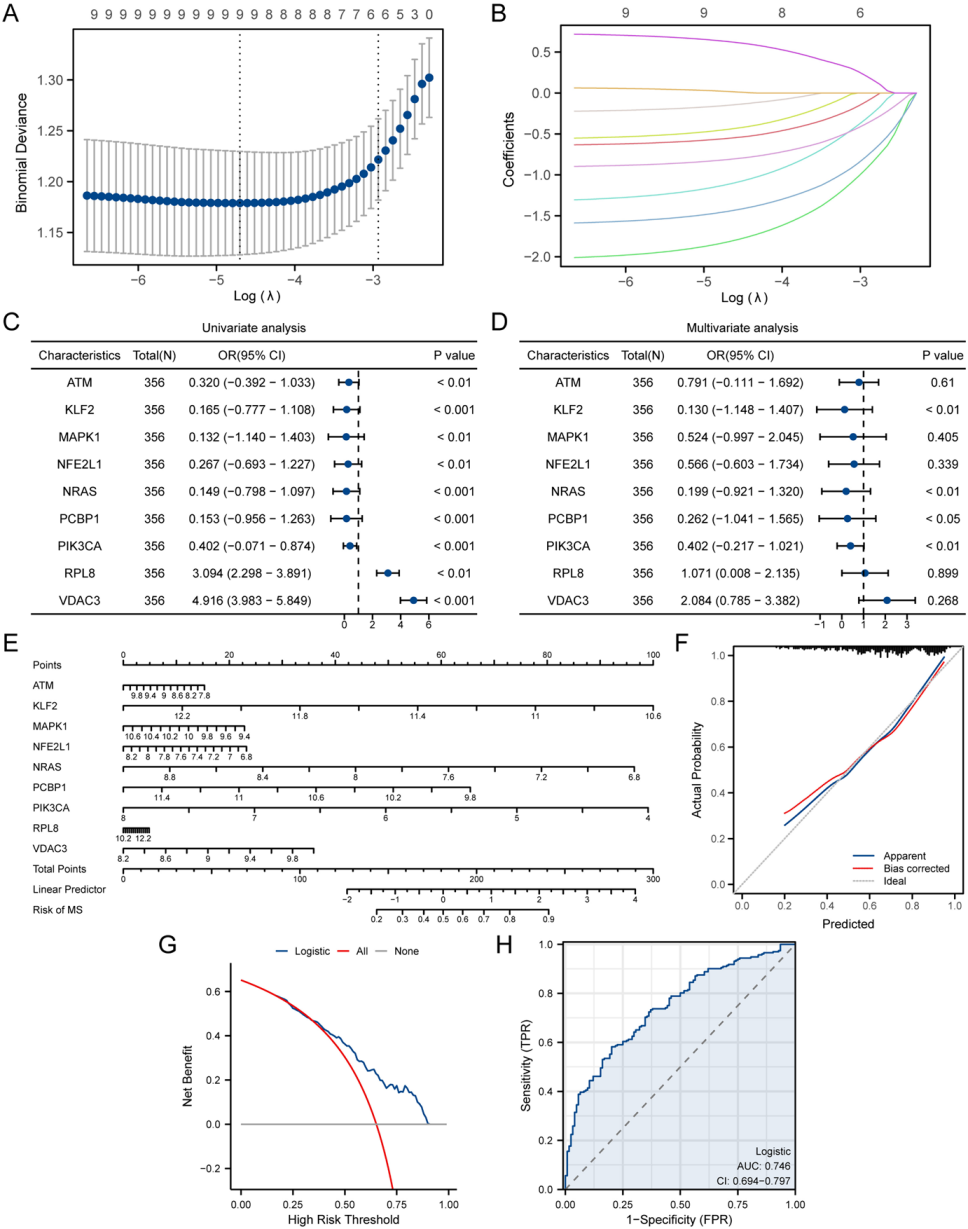
LASSO-Logistic regression analysis. **A** LASSO regression analysis of FRDEGs in the merged dataset. **B** Variable trajectory plot of the LASSO analysis. **C**–**D** Single/multiple factor logistic regression forest plot of 9 FRDEGs (*ATM, KLF2, MAPK1, NFE2L1, NRAS, PCBP1, PIK3CA, RPL8, VDAC3*). **E**–**H** Column chart (**E**), calibration curve plot (**F**), decision curve analysis plot (**G**), and ROC curve plot (**H**) of the logistic multiple factor regression model. AUC has low accuracy when it is between 0.5–0.7, moderate accuracy when it is between 0.7–0.9, and high accuracy when it is above 0.9

To investigate the diagnostic role of FRDEGs (*ATM, GSK3B, HMGCR, KLF2, MAPK1, NFE2L1, NRAS, PCBP1, PIK3CA, RPL8, VDAC3*) in MS, we first used LASSO regression analysis to build a diagnostic model of FRDEGs (Fig. 3A, B). As shown in Fig. 3A, B, after LASSO model selection, a total of 9 genes (*ATM, KLF2, MAPK1, NFE2L1, NRAS, PCBP1, PIK3CA, RPL8, VDAC3*) were included in the LASSO model. Next, we used logistic single and multiple factor regression analysis to create a forest plot of these 9 genes (Fig. 3C, D), the results showed that all 9 genes had significant (*p* < 0.05) diagnostic effects in logistic single factor regression analysis and were included in the multiple factor regression model. To further explore the joint diagnostic effect of the 9 FRDEGs on MS, we created a nomogram (Fig. 3E), calibration plot (Fig. 3F), decision curve analysis (DCA) plot (Fig. 3G) and ROC curve (Fig. 3H) based on the results of logistic multiple factor regression analysis. The results showed that logistic regression analysis of the 9 FRDEGs had a certain degree of diagnostic accuracy for MS.

### Multiple Sclerosis Patients can be Divided into Five Subtypes Based on the Expression of FRDEGs

**Fig. 4:**
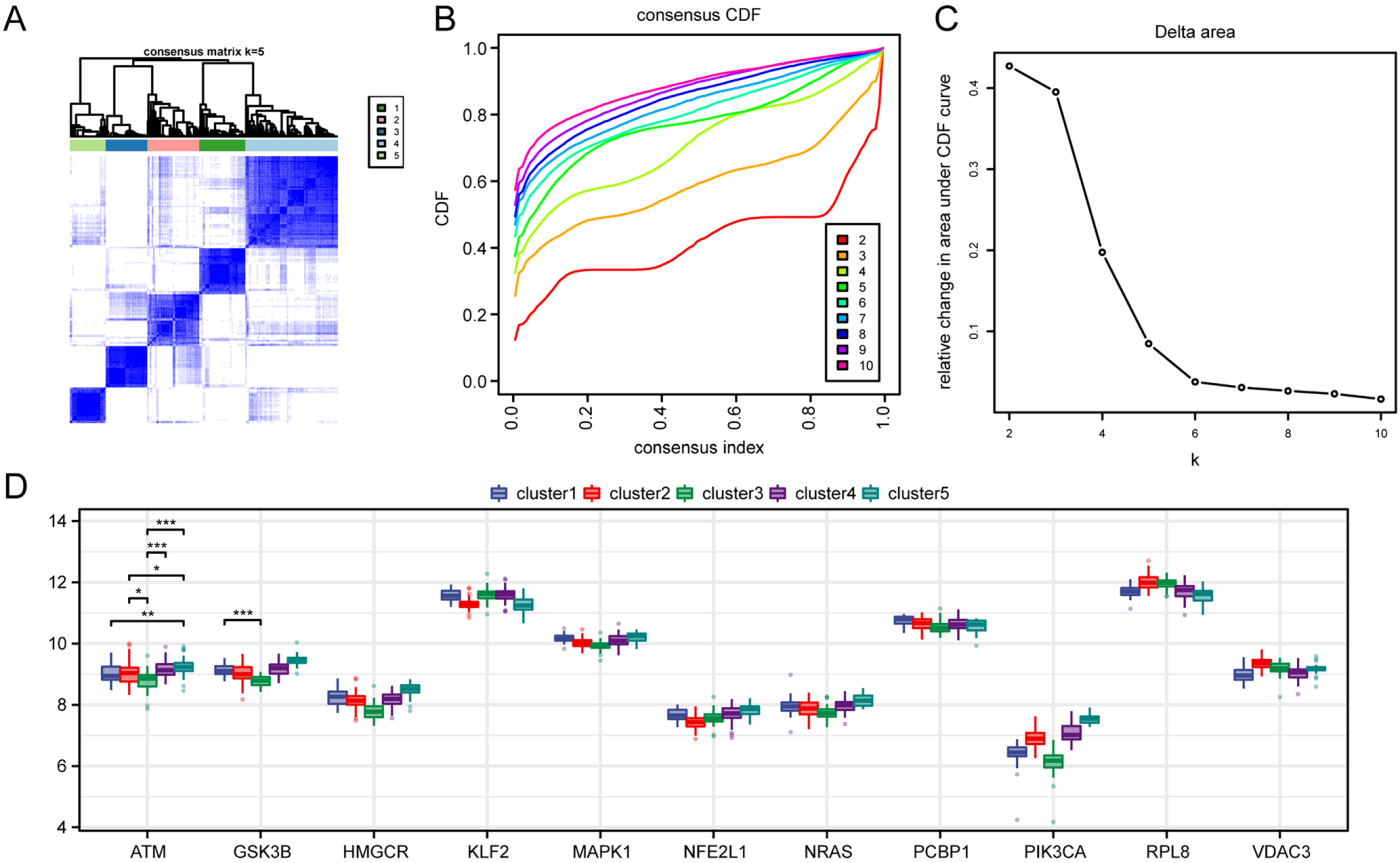
Molecular Subtype Analysis. **A**–**C** Consistency clustering heatmap (**A**), CDF plot (**B**), and Delta Area plot **C** of MS samples. **D** Grouped comparison plots of FRDEGs in different molecular subtypes (cluster1, cluster2, cluster3, cluster4, cluster5). The symbol * is equivalent to P < 0.05, indicating statistical significance, while the symbol *** is equivalent to P < 0.001, indicating high statistical significance

Based on the expression of FRDEGs (*ATM, GSK3B, HMGCR, KLF2, MAPK1, NFE2L1, NRAS, PCBP1, PIK3CA, RPL8, VDAC3*) in the merged dataset, we performed unsupervised consensus clustering on the merged dataset MS samples, which divided all MS group samples into 5 different subtypes (cluster 1: *n* = 40; cluster 2: *n* = 45; cluster 3: *n* = 36; cluster 4: *n* = 80; cluster 5: *n* = 31, Fig. 4A–C). Then, we used a group comparison map to show the expression differences of FRDEGs in different molecular subtypes (cluster 1, cluster 2, cluster 3, cluster 4, cluster 5) (Fig. 4D). The results showed that there were 2 FRDEGs (*ATM, GSK3B*) with significant (*p* < 0.05) expression differences in different molecular subtypes.

### There is an Interaction Relationship Between Ferroptosis-Related Proteins

We used the STRING database to analyze the protein–protein interactions of 11 FRDEGs (*ATM, GSK3B, HMGCR, KLF2, MAPK1, NFE2L1, NRAS, PCBP1, PIK3CA, RPL8, VDAC3*) and constructed proteinprotein interaction networks (PPI networks) for 7 of these FRDEGs. The interactions were visualized using the Cytoscape software (Figure S4A), and the scores of the FRDEGs under the MCC algorithm were analyzed using the cytoHubba plugin (Figure S4B). The specific scores are listed in Table 2.

**Table 2:** Top 7 FRDEGs ranked by MCC method

Rank	Name	Score
1	PIK3CA	8
1	MAPK1	8
3	NRAS	6
3	ATM	6
5	GSK3B	4
6	NFE2L1	1
6	KLF2	1

*FRDEGs* Ferroptosis-related differentially expressed genes, *MCC* Maximal Clique Centrality

We used the mRNA-miRNA data from the ENCORI and miRDB databases to predict miRNAs that interact with the 11 FRDEGs, and then visualized the interactions using the Cytoscape software (Figure S4C). The mRNA-miRNA interaction network consisted of 9 FRDEGs (*ATM, GSK3B, HMGCR, KLF2, MAPK1, NFE2L1, NRAS, PIK3CA, VDAC3*) and 72 miRNA molecules, with specific interaction relationships listed in Table S2. We searched for transcription factors (TFs) that bind to the FRDEGs using the CHIPBase and hTFtarget databases. After downloading and intersecting the interaction relationship data from both databases, we filtered for TFs sourced from blood and obtained interaction data for 10 FRDEGs (*ATM, GSK3B, HMGCR, KLF2, MAPK1, NFE2L1, PCBP1, PIK3CA, RPL8, VDAC3*) and 19 TFs, which were visualized using the Cytoscape software (Figure S4D). Specific mRNA-TF interaction relationships are listed in Table S3.

### The Function of Ferroptosis Related Protein in Multiple Sclerosis

**Fig. 5:**
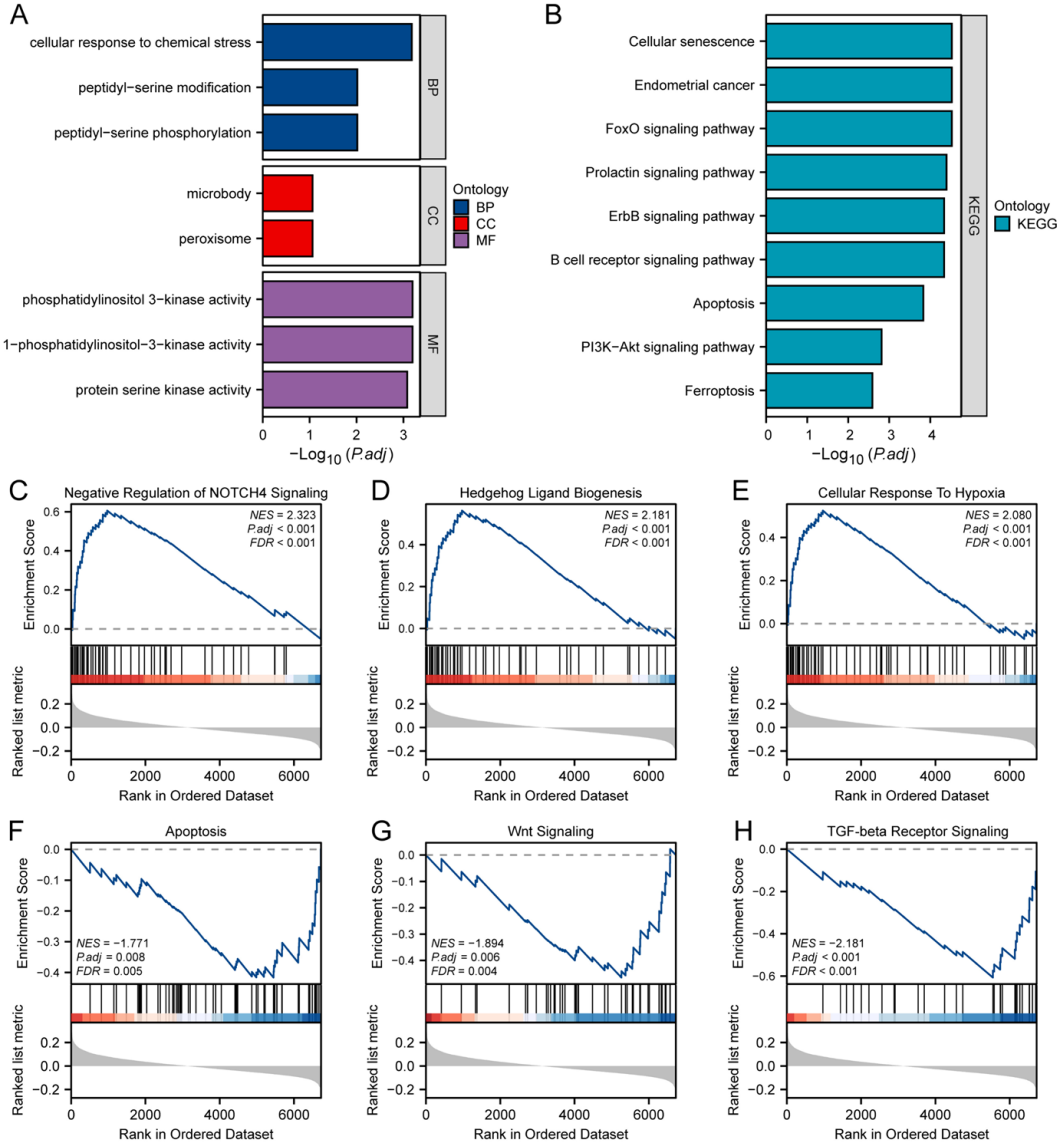
Enrichment analysis **A** Bar chart of GO functional enrichment analysis results for FRDEGs. **B** Bar chart of KEGG pathway enrichment analysis results for FRDEGs. **C**–**H** Significant enrichment of genes in Negative Regulation of NOTCH4 Signaling (**C**), Hedgehog Ligand Biogenesis (**D**), Cellular Response To Hypoxia (**E**), Apoptosis (**F**), Wnt Signaling (**G**), TGF-beta Receptor Signaling (**H**), and other pathways in the merged dataset. *FRDEGs* Ferroptosis-related Differentially Expressed Genes, *GO* Gene Ontology, *BP* biological process, *CC* cellular component, *MF* molecular function, *KEGG* Kyoto Encyclopedia of Genes and Genomes, *GSEA* Gene Set Enrichment Analysis

To explore the potential biological functions of FRDEGs, we performed analyses of biological processes, molecular functions, cellular components, and pathways for 11 FRDEGs (*ATM, GSK3B, HMGCR, KLF2, MAPK1, NFE2L1, NRAS, PCBP1, PIK3CA, RPL8, VDAC3*). The results showed that the differentially expressed genes were mainly enriched in biological processes such as cellular response to chemical stress, peptidyl-serine modification, and peptidyl-serine phosphorylation, as well as in cellular components such as microbodies and peroxisomes, and molecular functions such as phosphatidylinositol 3-kinase activity, 1-phosphatidylinositol-3-kinase activity, and protein serine kinase activity (Fig. 5A). We also conducted KEGG enrichment analysis of FRDEGs, which revealed that these genes were primarily enriched in pathways such as cellular senescence, endometrial cancer, FoxO signaling pathway, prolactin signaling pathway, ErbB signaling pathway, B cell receptor signaling pathway, apoptosis, PI3K-Akt signaling pathway, and ferroptosis (Fig. 5B). The pathway information obtained from GO/KEGG enrichment analysis is shown in Table 3.

**Table 3:** GOKEGG enrichment analysis results of FRDEGs

Ontology	ID	Description	GeneRatio	BgRatio	*p* value	*p*.adjust
BP	GO:0062197	Cellular response to chemical stress	5/11	332/18800	7.06e-07	0.0007
BP	GO:0018105	Peptidyl-serine phosphorylation	4/11	315/18800	2.33e-05	0.0096
BP	GO:0018209	Peptidyl-serine modification	4/11	338/18800	3.07e-05	0.0096
CC	GO:0005777	Peroxisome	2/11	141/19594	0.0027	0.0867
CC	GO:0042579	Microbody	2/11	141/19594	0.0027	0.0867
MF	GO:0016303	1-phosphatidylinositol-3-kinase activity	2/11	10/18410	1.46e-05	0.0006
MF	GO:0035004	Phosphatidylinositol 3-kinase activity	2/11	12/18410	2.14e-05	0.0006
MF	GO:0106310	Protein serine kinase activity	4/11	360/18410	4.26e-05	0.0008
KEGG	hsa04068	FoxO signaling pathway	5/10	131/8164	2.33e-07	2.97e-05
KEGG	hsa05213	Endometrial cancer	4/10	58/8164	4.67e-07	2.97e-05
KEGG	hsa04218	Cellular senescence	5/10	156/8164	5.57e-07	2.97e-05
KEGG	hsa04917	Prolactin signaling pathway	4/10	70/8164	1e-06	4.01e-05
KEGG	hsa01521	EGFR tyrosine kinase inhibitor resistance	4/10	79/8164	1.63e-06	4.59e-05
KEGG	hsa04012	ErbB signaling pathway	4/10	85/8164	2.19e-06	4.59e-05
KEGG	hsa04662	B cell receptor signaling pathway	4/10	82/8164	1.89e-06	4.59e-05
KEGG	hsa04210	Apoptosis	4/10	136/8164	1.43e-05	0.0001
KEGG	hsa04151	PI3K-Akt signaling pathway	4/10	354/8164	0.0005	0.0015
KEGG	hsa04216	Ferroptosis	2/10	41/8164	0.0010	0.0025

*GO* Gene Ontology, *KEGG* Kyoto Encyclopedia of Genes and Genomes, *FRDEGs* Ferroptosis-related differentially expressed genes, *BP* biological process, *CC* cellular component, *MF* molecular function

To determine the impact of gene expression levels on MS occurrence, we conducted Gene Set Enrichment Analysis (GSEA) to investigate the relationship between the gene expression in the combined dataset and the biological processes, cellular components, and molecular functions involve (Table 4). The results demonstrated that genes in the combined dataset were significantly enriched in pathways such as negative regulation of NOTCH4 signaling (Fig. 5C), hedgehog ligand biogenesis (Fig. 5D), cellular response to hypoxia (Fig. 5E), apoptosis (Fig. 5F), Wnt signaling (Fig. 5G), and TGF-beta receptor signaling (Fig. 5H).

**Table 4:** GSEA results of Merge dataset

ID	Set size	Enrichmentscore	NES	*p* value	*p*.adjust	*q* value
REACTOME_EUKARYOTIC_TRANSLATION_ELONGATION	75	0.7844969	3.318448	1e-10	6.15e-09	4.02e-09
KEGG_RIBOSOME	68	0.7917491	3.314182	1e-10	6.15e-09	4.02e-09
REACTOME_CELLULAR_RESPONSE_TO_STARVATION	108	0.7032205	3.098230	1e-10	6.15e-09	4.02e-09
REACTOME_NEGATIVE_REGULATION_OF_NOTCH4_SIGNALING	47	0.605887447	2.322749	6.67e-07	2.27e-05	1.48e-05
REACTOME_HEDGEHOG_LIGAND_BIOGENESIS	48	0.563514126	2.180714	1.05e-05	0.0002	0.0001
REACTOME_CELLULAR_RESPONSE_TO_HYPOXIA	55	0.524846627	2.079792	2.50e-05	0.0003	0.0002
KEGG_APOPTOSIS	59	-0.416467281	-1.770787	0.0010	0.0076	0.0049
WP_WNT_SIGNALING_PATHWAY	32	-0.488275265	-1.810595	0.0031	0.0169	0.0111
WP_WNT_SIGNALING	46	-0.464924798	-1.893947	0.0007	0.0055	0.0036
WP_TGFBETA_RECEPTOR_SIGNALING	29	-0.607093928	-2.180673	3.37e-05	0.0004	0.0003

*GSEA* Gene Set Enrichment Analysis

### The Ferroptosis-Related Protein Affects the Composition of Immune Cells in Multiple Sclerosis

**Fig. 6:**
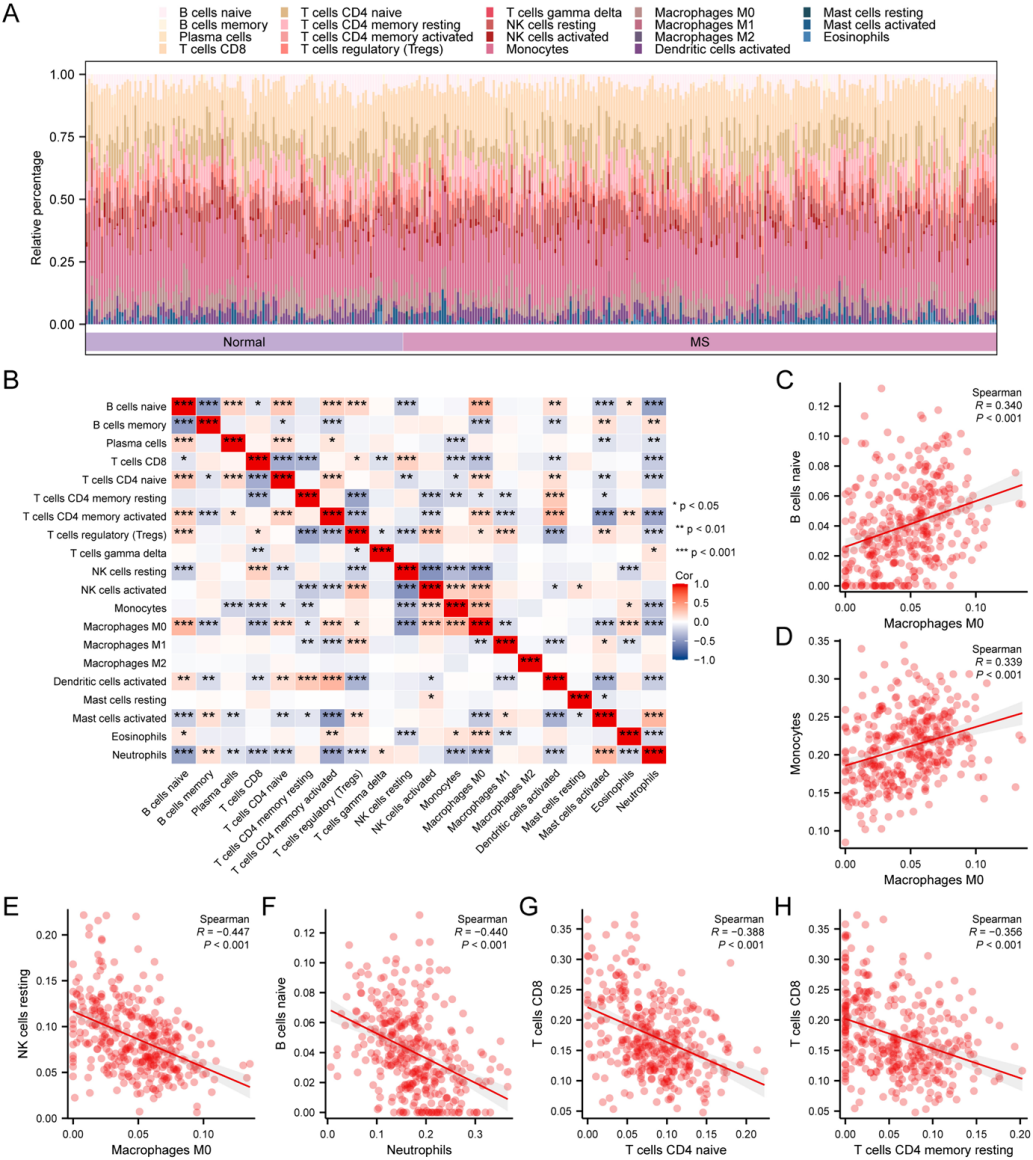
Immune cell infiltration analysis of merged datasets. **A** Stacked bar chart of 22 immune cells in different samples of the dataset, where differently colored bars represent different immune cells. **B** Correlation heatmap between the 22 immune cells. **C**–**H** Scatterplots showing the correlation between immune cells Macrophages M0 and B cells naive (**C**), Macrophages M0 and Monocytes (**D**), Macrophages M0 and NK cells resting (**E**), Neutrophils and B cells naive (**F**), T cells CD4 naive and T cells CD8 (**G**), and T cells CD4 memory resting and T cells CD8 (**H**). The symbol * is equivalent to p < 0.05 and has statistical significance; the symbol ** is equivalent to p < 0.01 and has high statistical significance; the symbol *** is equivalent to p < 0.001 and has extremely high statistical significance. The correlation coefficient r is positive, indicating a possible positive correlation between two variables, and negative, indicating a possible negative correlation between two variables. A correlation coefficient absolute value above 0.8 is a strong correlation, between 0.5–0.8 is a moderate correlation, between 0.3–0.5 is a general correlation, and below 0.3 is a weak or no correlation

We utilized the CIBERSORTx algorithm to calculate the infiltration levels of 22 immune cell types in different samples, which were displayed in a stacked bar graph (Fig. 6A). We also performed correlation analysis on these 22 immune cell types and presented the results as a correlation heatmap (Fig. 6B). We selected several pairs of cells with the highest absolute correlation coefficients and plotted them in scatter plots (Fig. 6C–H). The results showed a significant positive correlation (*p* < 0.05) between Macrophages M0 and B cells naive (Fig. 6C, r = 0.340), as well as between Macrophages M0 and Monocytes (Fig. 6D, r = 0.339). In contrast, a significant negative correlation (p < 0.05) was observed between Macrophages M0 and NK cells resting (Fig. 6E, r = − 0.447), Neutrophils and B cells naive (Fig. 6F, r = − 0.440), T cells CD4^+^ naive and T cells CD8^+^ (Fig. 6G, r = − 0.388), and T cells CD4^+^ memory resting and T cells CD8^+^ (Fig. 6H, r = − 0.356).

### The Ferroptosis-Related Protein can Classify Multiple Sclerosis Patients into Different Immune Subtypes

**Fig. 7:**
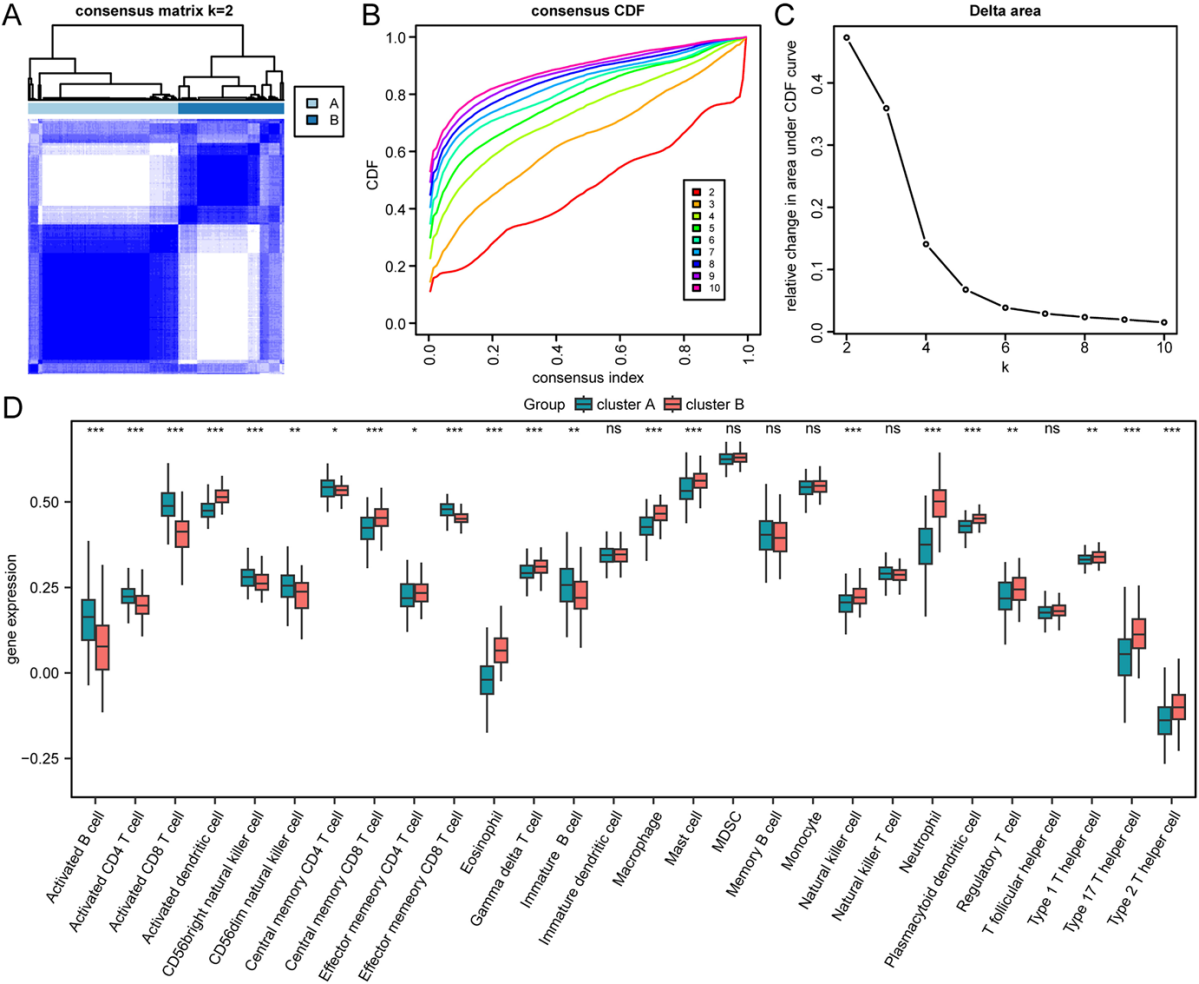
Immune subtyping analysis. Consensus clustering (k = 2) of immune infiltration matrix results. **B**–**C** Cumulative distribution function (CDF) plot for different numbers of clusters in the consensus clustering (**B**) and Delta plot for CDF curve (**C**). **D** Comparison of gene expression levels of different immune cells in two clusters. Horizontal axis represents cells and vertical axis represents levels of infiltration. Blue-green represents cluster A, and orange-red represents cluster B. The symbol "ns" is equivalent to p > 0.05, not statistically significant; the symbol * is equivalent to p < 0.05, statistically significant; the symbol ** is equivalent to p < 0.01, highly statistically significant; and the symbol *** is equivalent to p < 0.001, extremely statistically significant

**Fig. 8:**
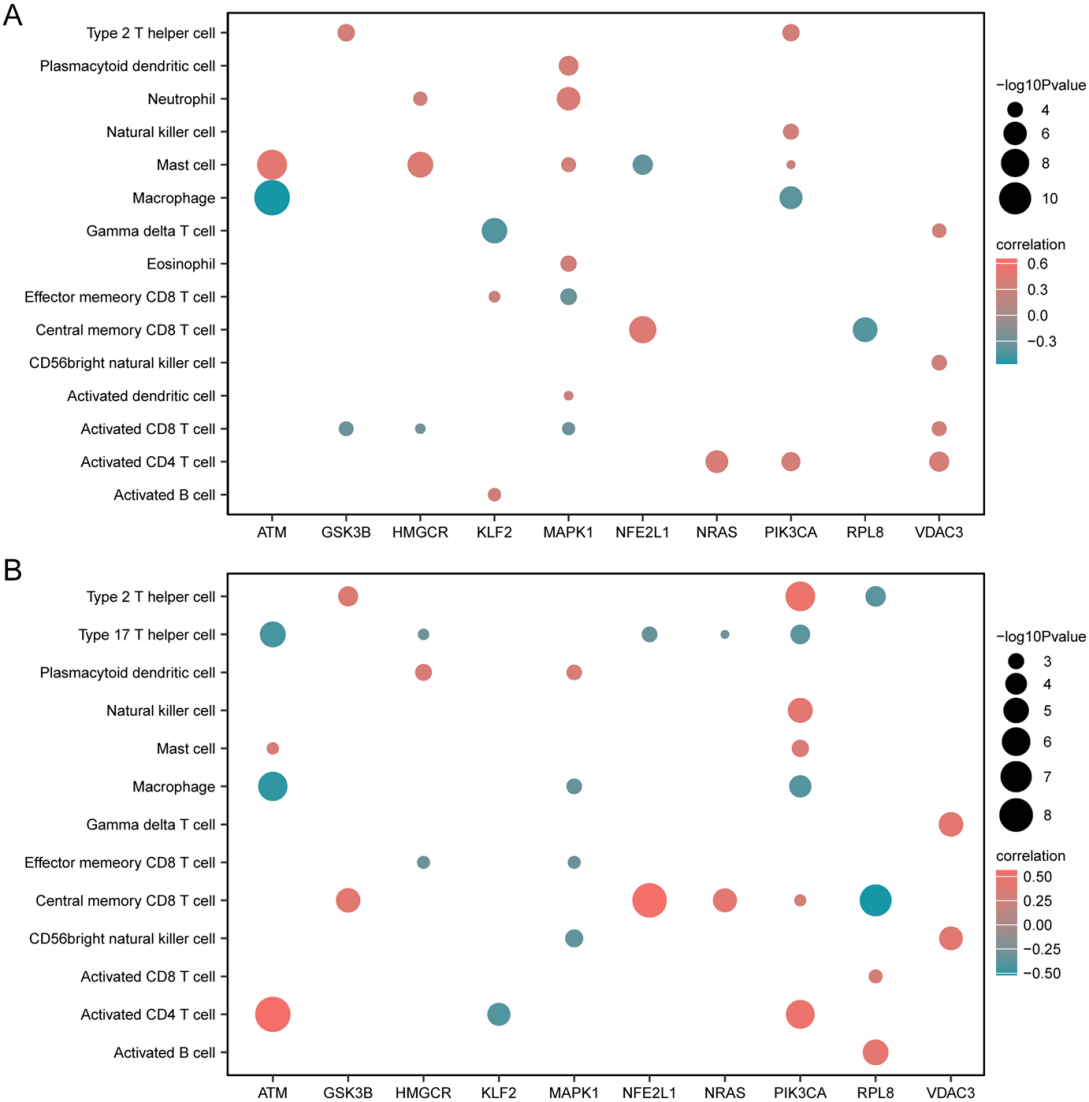
Correlation analysis of immune feature subtypes. **A**, **B** Heatmap showing the correlation between immune cell infiltration abundance in cluster A (**A**) and cluster B (**B**) and FRDEGs (Ferroptosis-related Differentially Expressed Genes)

After organizing the expression profile data of the merged dataset, we used the ssGSEA algorithm to calculate the infiltration status of 28 immune cells. Then, based on the infiltration abundance of the 28 immune cells, we performed unsupervised consensus clustering on the MS group samples of the merged dataset, dividing all MS group samples into two different subtypes (cluster A: *n* = 136; cluster B: *n* = 96, Fig. 7A–C). Next, we used a group comparison plot to show the differences in immune cell infiltration between different immune feature subtypes (cluster A, cluster B) (Fig. 7D). According to Fig. 7D, there were 16 immune cells (Activated B cell, Activated CD4^+^ T cell, Activated CD8^+^ T cell, Activated dendritic cell, CD56^+^ bright natural killer cell, Central memory CD8^+^ T cell, Effector memory CD8^+^ T cell, Eosinophil, Gamma delta T cell, Macrophage, Mast cell, Natural killer cell, Neutrophil, Plasmacytoid dendritic cell, Type 17 T helper cell, Type 2 T helper cell) that had significant (*p* < 0.05) differences in infiltration between different immune feature subtypes (cluster A,clusterB).In addition, we also generated correlation heatmaps (Fig. 8A, B) showing significant (*p* < 0.05) associations between immune cells and FRDEGs in different subtypes (cluster A, cluster B). As shown in the figures, *ATM* had a significant negative correlation with Macrophage in both subtypes, and had a significant positive correlation with Activated CD4^+^ T cell in cluster B. *NFE2L1* had a significant positive correlation with Central memory CD8^+^ T cell in cluster B.

### The Ferroptosis-Related Protein can Diagnose Multiple Sclerosis Separately

**Fig. 9:**
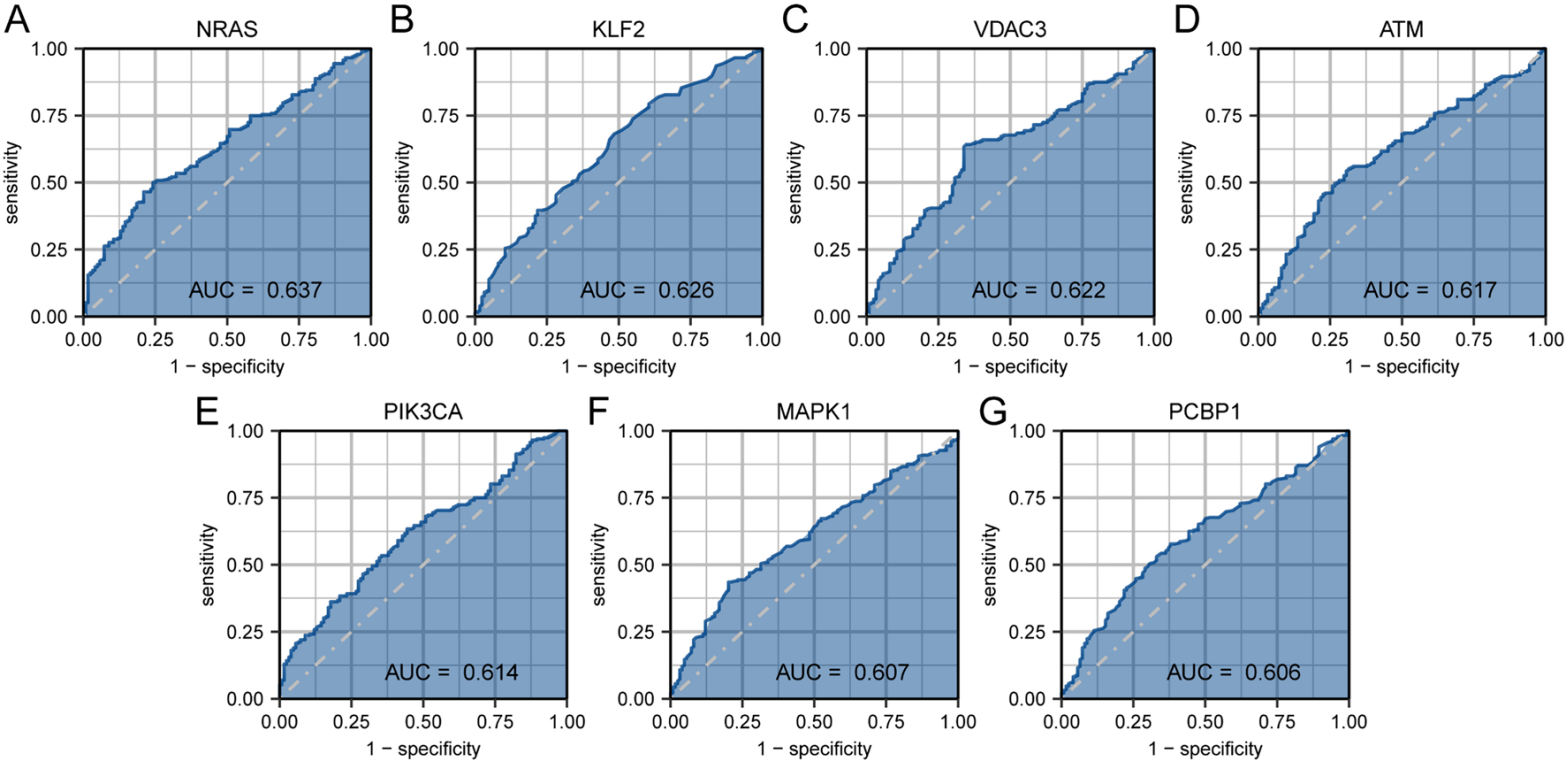
ROC validation of FRDEGs. **A**–**G** ROC validation of *NRAS* (**A**), *KLF2* (**B**), *VDAC3* (**C**), *ATM* (**D**), *PIK3CA* (**E**), *MAPK1* (**F**), and *PCBP1* (**G**) in the combined dataset. The closer the AUC is to 1, the better the diagnostic performance. AUC has low accuracy in the range of 0.5–0.7, moderate accuracy in the range of 0.7–0.9, and high accuracy above 0.9

To investigate the diagnostic performance of 11 FRDEGs (*ATM, GSK3B, HMGCR, KLF2, MAPK1, NFE2L1, NRAS, PCBP1, PIK3CA, RPL8, VDAC3*), we performed ROC validation of these 11 FRDEGs in the merged dataset, and selected genes with an AUC greater than 0.6 for visualization (Fig. 9A-G). Among them, *NRAS* (Fig. 9A, AUC = 0.637), *KLF2* (Fig. 9B, AUC = 0.626), *VDAC3* (Fig. 9C, AUC = 0.622), *ATM* (Fig. 9D, AUC = 0.617), *PIK3CA* (Fig. 9E, AUC = 0.614), *MAPK1* (Fig. 9F, AUC = 0.607), and *PCBP1* (Fig. 9G, AUC = 0.606) showed low diagnostic accuracy in distinguishing MS and Normal groups.

## Discussion

While MS is not a purely genetic disease, certain genes could impact its occurrence (Axisa and Hafler 2016; Sun et al. 2020). In fact, iron death plays an important role in the pathogenesis of multiple sclerosis, as there is a significant amount of iron and iron metabolism abnormalities in the brains, spinal cords, and other neurons of multiple sclerosis patients, leading to iron metabolism imbalance within cells and subsequently triggering iron death (Luoqian et al. 2022; Katz Sand 2015). We used bioinformatics methods to screen iron death-related genes related to the diagnosis of multiple sclerosis and established a diagnostic model. Tests showed that this diagnostic model could accurately diagnose patients’ conditions. We then performed subgroup analysis based on the expression of iron death-related genes and divided patients into high-expression and low-expression groups. The results showed differences in immune function and immune cell infiltration between the two groups. Our study not only confirms the correlation between iron death and multiple sclerosis but also demonstrates the diagnostic value of iron death-related genes in multiple sclerosis. This provides guidance for clinical practice and direction for mechanism research.

In our study, we first screened differentially expressed genes related to iron death in the multiple sclerosis dataset, including *ATM, GSK3B, HMGCR, KLF2, MAPK1, NFE2L1, NRAS, PCBP1, PIK3CA, RPL8, and VDAC3*, among others. Several of these molecules have been reported to be associated with the occurrence of multiple sclerosis. Aberrant expression or mutation of these genes might be related to the onset and progression of multiple sclerosis, suggesting a close correlation between iron death-related genes and multiple sclerosis (Kuhlmann et al. 2023a, b). *VDAC3*, one of the nuclear genes of mitochondrial, is the most significantly up-regulated iron death-related gene (Yang et al. 2020). Its main function is to serve as a water channel protein in the mitochondrial membrane. This protein is expressed in various tissues such as liver, muscle, and brain, and is considered as a key regulator of mitochondrial cell death pathways. In previous studies, *VDAC3* has been implicated in various diseases such as cancer, diabetes, and neurological disorders (Reina et al. 2016). Recent research suggests that *VDAC3* is a critical regulator of ferroptosis (Huang et al. 2022; Reina et al. 2016). The expression of *VDAC3* increases with the concentration of iron ions, and its expression is significantly increased in a state of iron overload. The protein it encodes is PI3K p110α, a protein that is believed to be involved in the origin and development of various human tumors. Recent studies have shown that the expression of *PIK3CA* (Phosphatidylinositol-4, 5-bisphosphate 3-kinase catalytic subunit alpha) is upregulated in patients with multiple sclerosis (Hong et al. 2022; Canaud et al. 2021). This might be due to the critical regulatory role that *PIK3CA* plays in both normal and pathological inflammatory responses, as well as its impact on cell gene expression (Canaud et al. 2021). Additionally, iron overload might impact the occurrence and development of neurodegenerative diseases through the activation of the PI3K/Akt signaling pathway. This suggests that *PIK3CA* might play a critical role in neuronal death caused by intracellular iron ion accumulation (Canaud et al. 2021). The significant upregulation of *PIK3CA* might be a disease signal that is related to iron death pathways and plays a crucial role in the onset and progression of multiple sclerosis (Venot and Canaud 2017). This suggests that *PIK3CA* might have different mechanisms of action in the onset and progression of different neurological diseases. For patients with multiple sclerosis, treatments targeting *PIK3CA* might be more effective.

Currently, we have used single genes to predict the prognosis of patients and found that both *NRAS* and *KLF2* are good diagnostic genes for multiple sclerosis, and they could independently predict the onset of multiple sclerosis. *NRAS* is a common human oncogenic mutation gene, and previous studies have reported its close relationship with the occurrence and development of various malignancies (Randic et al. 2021). *NRAS* is a member of the RAS family and a crucial signaling protein kinase (Jenkins and Sullivan 2016). Under normal physiological conditions, *NRAS* could participate in biological processes such as cell proliferation, differentiation, and apoptosis, and play an important regulatory role in these processes (Ye et al. 2019; Messina 2020). Recent studies have shown that abnormal activation of *NRAS* in MS patients could cause abnormal proliferation and activation of various immune cells, leading to severe inflammatory and autoimmune responses (Koch-Henriksen and Magyari 2021). Another key gene, *KLF2*, is a transcription factor that plays an important role in regulating cell migration, proliferation, and differentiation (Levite 2016; Jiang et al. 2020). *KLF2* continues to play an important role in immune cells, such as regulating the differentiation and function of T cells and the cytotoxic activity of B cells (Tang et al. 2022). For the pathogenesis of MS, studies have found that *KLF2* might play an important role in T cell differentiation and *VEGFR2* expression regulation (Lu et al. 2021; Jiang et al. 2020). Hence, we could explore the potential mechanism and application prospects of *KLF2* in disease diagnosis and treatment.

Based on the known literature and existing analysis results, FRDEGs might play an important regulatory role in multiple sclerosis, participating in the regulation of multiple biological processes and pathways. For example, some signaling molecules of cell apoptosis, such as Cytochrome C and death receptor Fas, can be used as serum biomarkers, and might have guiding significance for early diagnosis and treatment of MS and other neurodegenerative diseases (Santucci et al. 2019; Jiang et al. 2020). The Wnt pathway is a signaling pathway widely present in animals, plants, and other organisms (Santucci et al. 2019). It could regulate various biological processes such as cell proliferation, differentiation, and apoptosis by controlling gene expression (Santucci et al. 2019). The pathway is mainly composed of various factors, including Wnt protein, Frizzled receptor and ligand, Disheveled protein, GSK-3β enzyme, and β-catenin protein. In the resting state, β-catenin is phosphorylated by GSK-3β enzyme, discarded, and marked as invalid. When the Wnt signal acts on the Frizzled receptor and ligand of the cell, Disheveled protein is phosphorylated, leading to the inhibition of GSK-3β enzyme (Demuro et al. 2021). The β-catenin protein is no longer phosphorylated and enters the nucleus to bind with transcription factors *LEF/TCF*, triggering the expression of Wnt genes and regulating various cellular functions (Liu et al. 2022; Kennedy et al. 2022). Research has shown that the Wnt pathway plays an important role in the development and repair of the central nervous system. For example, under live-cell microscopy, it has been observed that the Wnt signaling pathway could promote T-cell proliferation by suppressing the expression of *Foxp3*, thereby enhancing the autoimmune response (Rim et al. 2022). The study also found that activation of the Wnt pathway could inhibit Th17 cells, promote the differentiation of regulatory T cells, and therefore has important therapeutic value in the treatment of MS (Russell and Monga 2018). In conclusion, the potential biological functions of FRDEGs in multiple sclerosis are highly complex, involving the regulation of multiple signaling pathways and biological processes. This provides important references for a better understanding of the mechanisms underlying the occurrence and development of multiple sclerosis.

In multiple sclerosis, the degree of immune cell infiltration is associated with the onset and progression of the disease (Dobson and Giovannoni 2019). We calculated the correlation of immune cells in multiple sclerosis. The results showed a positive correlation between Macrophages M0 and B cells naive, and between Macrophages M0 and Monocytes among the 22 types of immune cells, suggesting that these immune cells might participate in the pathological process of multiple sclerosis together (Vogel et al. 2014). Macrohpages M0 and NK cells resting, neutrophils and B cells naive, T cells CD4^+^ naive and CD8^+^ T cells, and CD4^+^ T cells memory resting and CD8^+^ T cells show negative correlation, which might suggest a competitive or regulatory relationship among these immune cells. In our research, we discovered that the most relevant cells to multiple sclerosis are macrophages M0 and B cells naive. Recent studies have indicated that M0 macrophages participate in the immunopathology of MS (Dobson and Giovannoni 2019). Specifically, M0 macrophages regulate the body’s immune system by producing various cytokines and mediators, thereby playing an important role in the onset of MS. On one hand, M0 macrophages could recognize and eliminate foreign pathogens and induce cell toxicity during the process, while also being responsible for defending against exogenous and endogenous injuries and viral infections. Meanwhile, various cytokines produced by M0 macrophages such as IL-10 and TGF-α could regulate the immune system in the body, helping to inhibit the development and progression of MS inflammatory response and protect neurons from damage (Niino et al. 2009). On the other hand, it has been found that M0 macrophages also increase in most MS patients. This might reflect an increase in M0 macrophage activity and open antigen presentation, possibly related to their regulatory role in lesions and further initiation of immune responses (Duddy et al. 2007). We then performed calculations and clustering analyses on the infiltration of 28 types of immune cells in MS samples, dividing all MS group samples into two different subtypes (cluster A and cluster B). Through the group comparison graph, we found significant differences in the infiltration of 16 types of immune cells in these two subtypes. Among them, activated CD8^+^ T cells play an important role as one of the CD8^+^ T cell populations in the immune response of MS. Studies have shown that in MS, CD8^+^ T cells recognize and attack neurons expressing antigens through CD8^+^ T cells mediated recognition of *MHC-I* molecules, triggering an immune response and tissue damage. In addition, CD8^+^T cells could also produce cytokines, further stimulating and promoting neuronal damage and autoimmune responses (Mittrücker et al. 2014; Rodríguez Murúa et al. 2022a). Therefore, we need to pay attention to the role of CD8^+^T cells in multiple sclerosis and the potential prospect of intervening these cells in treatment.

However, our study inevitably has some limitations. Firstly, the sample size of this study was not large enough, and further larger MS cohort studies are needed to further confirm and extend the conclusions of this study. Secondly, this study is the second mining and analysis of previously published datasets, so we do not know the severity of the patients, treatment methods and related clinical data, the lack of clinically relevant data might be a confounding factor in our analysis. Thirdly, all our samples are from peripheral blood. Due to limitations of reagents and operations, the special values in our prediction model are not representative. Fourthly, our study is all composed of bioinformatics analysis methods and statistical methods, without experimental validation of FRDEGs. Therefore, in future studies, we will improve the experimental design to make the analysis more convincing.

## Conclusions

In conclusion, there is a close relationship between ferroptosis and multiple sclerosis, and further research is expected to reveal the underlying mechanisms and provide new ideas and methods for the treatment of this disease. We established a diagnostic model based on ferroptosis-related genes using bioinformatics methods, and the test showed that the model could accurately diagnose the patients’ condition. Subgroup analysis further confirmed the diagnostic value of ferroptosis-related genes in multiple sclerosis, and revealed the impact of different gene expression levels on the patients’ immune function and immune cell infiltration. This study not only strengthens the correlation between ferroptosis and multiple sclerosis, but also provides practical guidance for clinical practice and important directions for further research on the disease mechanism.

## Supplementary Information

Below is the link to the electronic supplementary material.

Supplementary file1 (TIF 950 KB)—Figure S1 Flowchart

Supplementary file2 (TIF 6319 KB)—Figure S2 Dataset merging and correction. (A) Boxplot of merged dataset before correction. (B)Boxplot of merged dataset after correction. (C)Principal Component Analysis (PCA) plot of merged dataset before correction. (D)PCA plot of merged dataset after correction

Supplementary file3 (TIF 2773 KB)—Figure S3 Differential analysis of merged datasets. (A)Volcano plot of differentially expressed genes (DEGs) between MS and Normal groups in the merged dataset. (B-C) Venn diagrams showing the intersection of DEGs and ferroptosis-related genes (FRGs) in the merged dataset. (D)Volcano plot of DEGs between MS and Normal groups in the validation dataset GSE21942. E-F. Venn diagrams showing the intersection of ferroptosis-related DEGs (FRDEGs) in the merged dataset and DEGs in the validation dataset GSE21942

Supplementary file4 (TIF 4763 KB)—Figure S4 Interaction Network of FRDEGs. Protein-protein interaction network (PPI Network) of FRDEGs. Network graph of scores correlated with FRDEGs under the MCC algorithm. The color of rectangles from yellow to red represents gradually increasing scores. (C-D) mRNA-miRNA (C) and mRNA-TF (D) interaction network of FRDEGs. Green diamonds indicate mRNA, blue diamonds indicate miRNA, and blue ellipses indicate transcription factors (TF). PPI Network: Protein-protein Interaction Network. FRDEGs: Ferroptosis-related Differentially Expressed Genes. MCC: Maximal Clique Centrality. TF: Transcription factors

Supplementary file5 (DOCX 17 KB)

Supplementary file6 (DOCX 15 KB)

Supplementary file7 (DOCX 12 KB)

## Data Availability

The datasets used and analyzed in the present study are available from the corresponding author on reasonable request.

## Abbreviations

AUC: Area under curve
BH: Benjamini–Hochberg
BP: Biological process
CC: Cellular component
CDF: Cumulative distribution function
CNS: Central nervous system
DCA: Decision curve analysis
DEGs: Differentially expressed genes
FRGs: Ferroptosis-related genes
FRDEGs: Ferroptosis-related differentially expressed genes
GSEA: Gene set enrichment analysis
GO: Gene ontology
KEGG: Kyoto encyclopedia of genes and genomes
LASSO: Least absolute shrinkage and selection operator
MF: Molecular function
MS: Multiple sclerosis
MSigDB: Molecular signatures database
PCA: Principal component analysis
PPI: Protein–protein interaction
ROC curve: Receiver operating characteristic curve
TF: Transcription factor
LASSO: Least absolute shrinkage and selection operator
MCC: Maximal clique centrality
KEGG: Kyoto encyclopedia of genes and genomes
ssGSEA: Single sample gene set enrichment analysis
