# Supplementary figures and images for: Establishment of the Diagnostic Signature of Ferroptosis Genes in Multiple Sclerosis
Source: Biochem Genet. 2024 Jun 17;63(4):3065–94. doi: 10.1007/s10528-024-10832-3 (PMC12271295; doi:10.1007/s10528-024-10832-3)

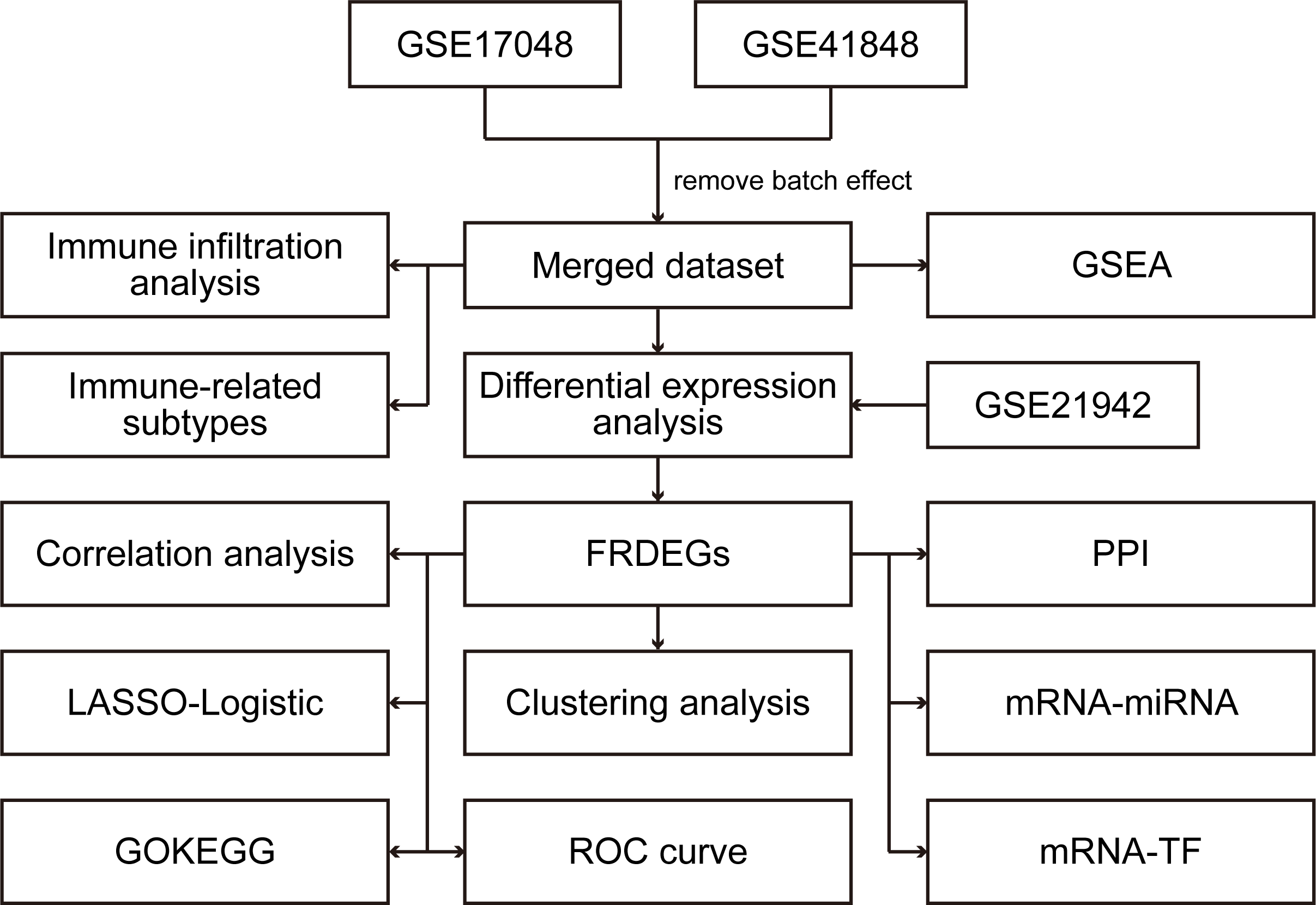

Supplement: Supplementary file 1 — Supplementary file1 (TIF 950 KB)—Figure S1 Flowchart [file 10528_2024_10832_MOESM1_ESM.tif]

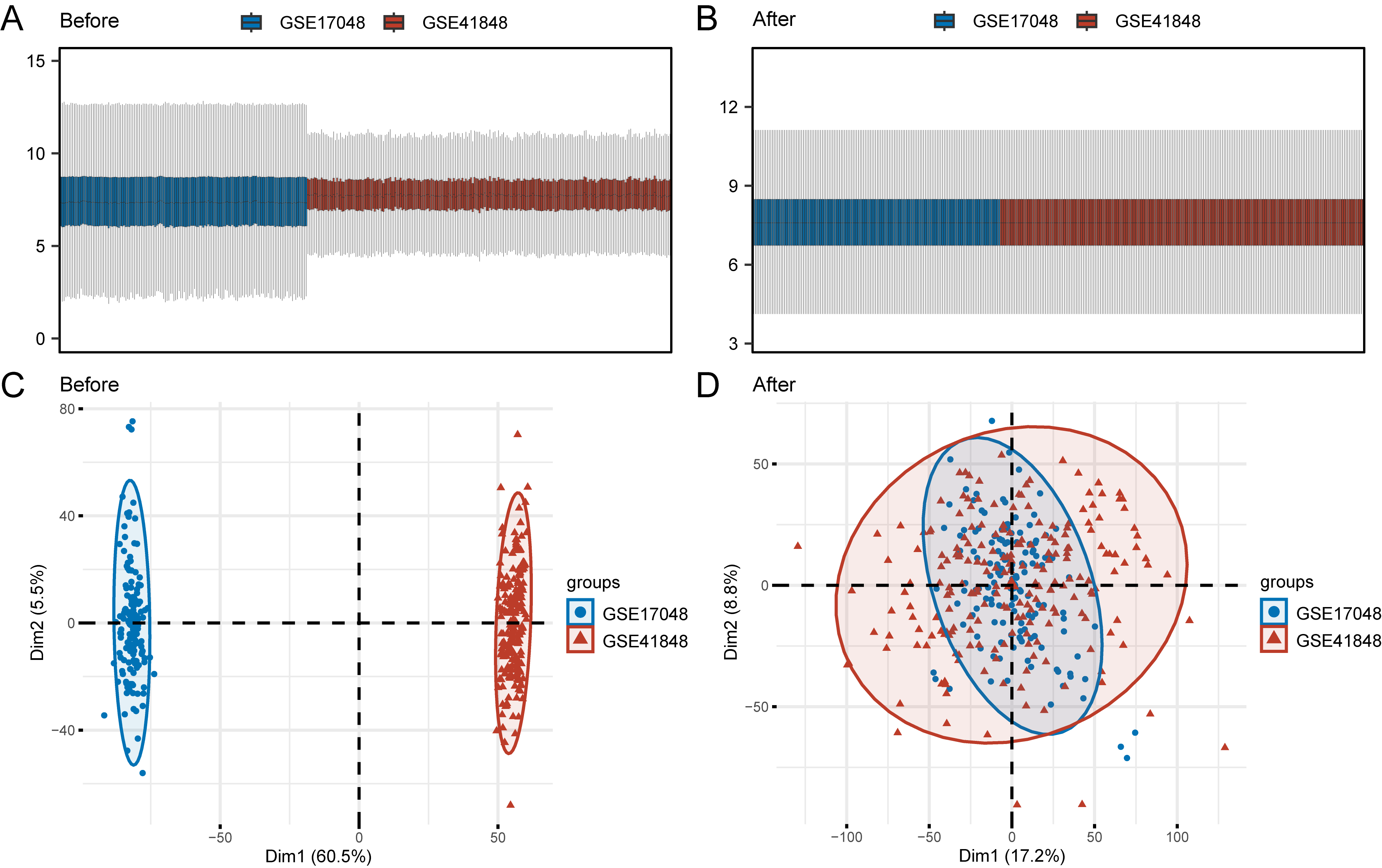

Supplement: Supplementary file 2 — Supplementary file2 (TIF 6319 KB)—Figure S2 Dataset merging and correction. (A) Boxplot of merged dataset before correction. (B)Boxplot of merged dataset after correction. (C)Principal Component Analysis (PCA) plot of merged dataset before correction. (D)PCA plot of merged dataset after correction [file 10528_2024_10832_MOESM2_ESM.tif]

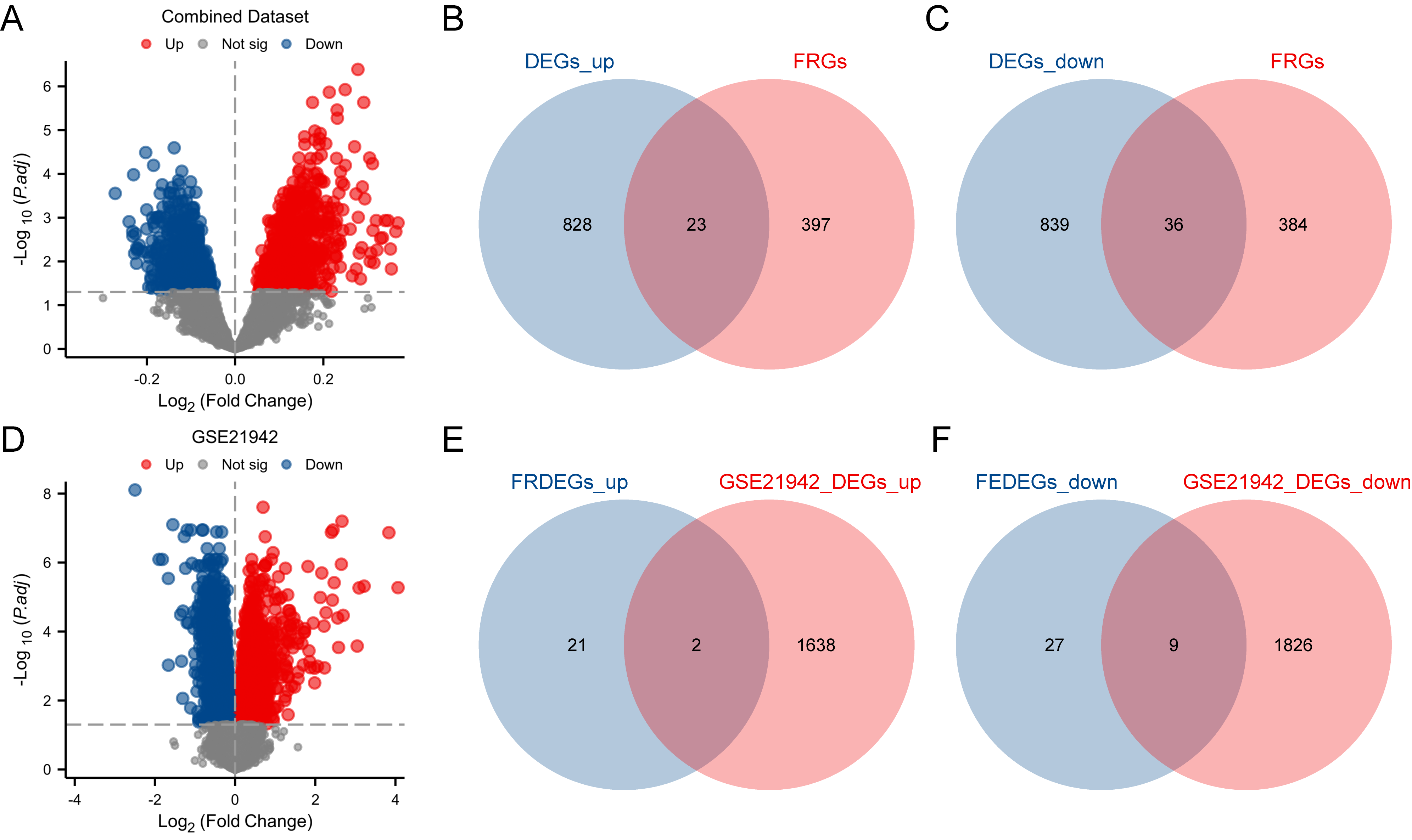

Supplement: Supplementary file 3 — Supplementary file3 (TIF 2773 KB)—Figure S3 Differential analysis of merged datasets. (A)Volcano plot of differentially expressed genes (DEGs) between MS and Normal groups in the merged dataset. (B-C) Venn diagrams showing the intersection of DEGs and ferroptosis-related genes (FRGs) in the merged dataset. (D)Volcano plot of DEGs between MS and Normal groups in the validation dataset GSE21942. E-F. Venn diagrams showing the intersection of ferroptosis-related DEGs (FRDEGs) in the merged dataset and DEGs in the validation dataset GSE21942 [file 10528_2024_10832_MOESM3_ESM.tif]

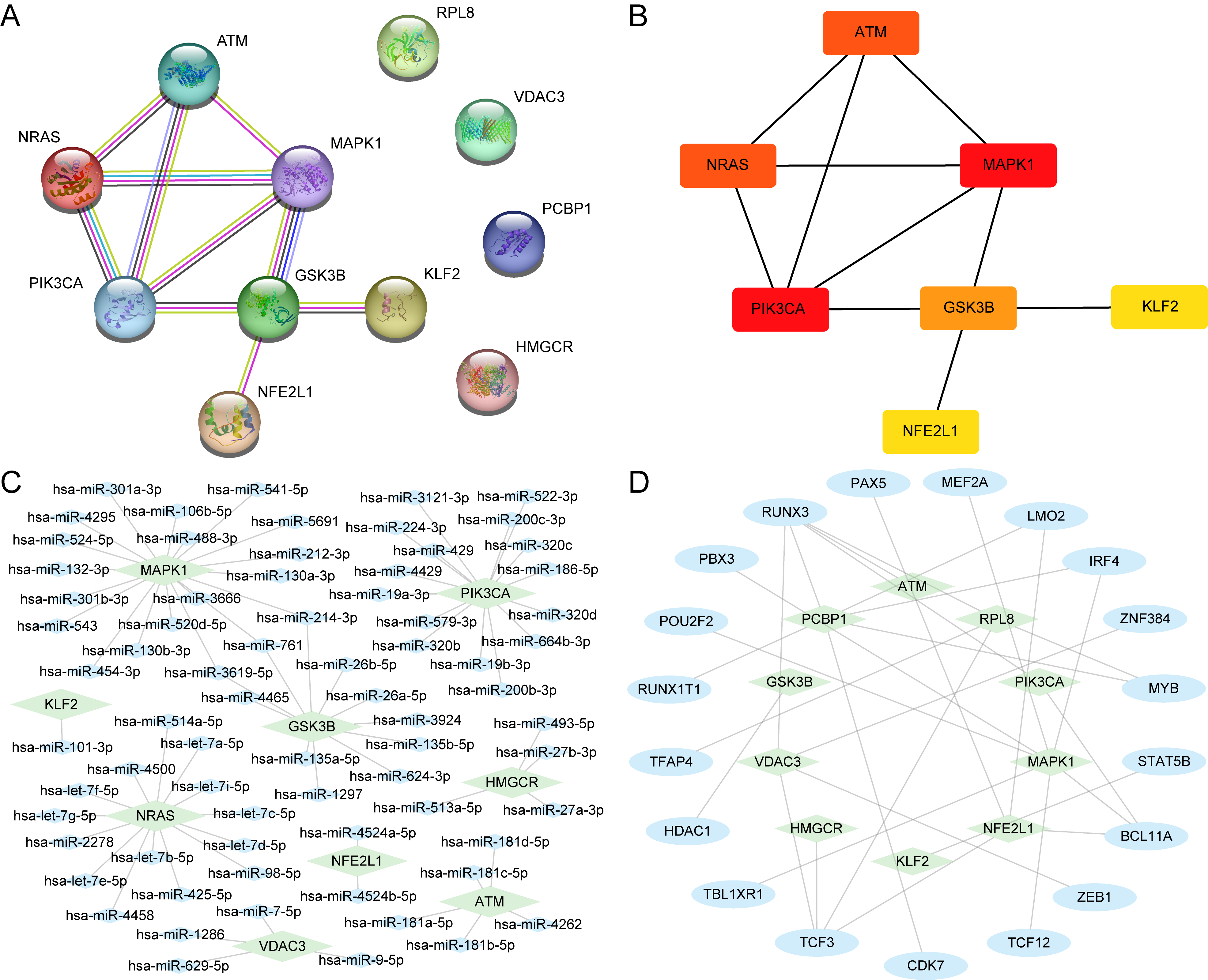

Supplement: Supplementary file 4 — Supplementary file4 (TIF 4763 KB)—Figure S4 Interaction Network of FRDEGs. Protein-protein interaction network (PPI Network) of FRDEGs. Network graph of scores correlated with FRDEGs under the MCC algorithm. The color of rectangles from yellow to red represents gradually increasing scores. (C-D) mRNA-miRNA (C) and mRNA-TF (D) interaction network of FRDEGs. Green diamonds indicate mRNA, blue diamonds indicate miRNA, and blue ellipses indicate transcription factors (TF). PPI Network: Protein-protein Interaction Network. FRDEGs: Ferroptosis-related Differentially Expressed Genes. MCC: Maximal Clique Centrality. TF: Transcription factors [file 10528_2024_10832_MOESM4_ESM.tif]
