# Supplementary material for: Establishment of the Diagnostic Signature of Ferroptosis Genes in Multiple Sclerosis
Source: Biochem Genet. 2024 Jun 17;63(4):3065–94. doi: 10.1007/s10528-024-10832-3 (PMC12271295; doi:10.1007/s10528-024-10832-3)
Supplement: Supplementary file 5 — Supplementary file5 (DOCX 17 KB) [file 10528_2024_10832_MOESM5_ESM.docx]

**Table S1. Ferroptosis-related genes**

| Gene Symbol | | | | | |
| --- | --- | --- | --- | --- | --- |
| RPL8 | CYP4F8 | MIB1 | CHMP5 | STEAP3 | HCAR1 |
| IREB2 | MLLT1 | AKR1C1 | CHMP6 | PCBP2 | BMAL1 |
| ATP5MC3 | TTPA | AKR1C2 | MTF1 | ATG5 | RETREG1 |
| CS | GRIA3 | AKR1C3 | COPZ1 | ATG7 | YY1 |
| EMC2 | POM121L12 | MUC1 | NUPR1 | HMGB1 | SPHK1 |
| ACSF2 | AEBP2 | NFE2L2 | USP35 | SP1 | EFEMP1 |
| NOX1 | AGPS | SLC40A1 | HSF1 | PANX1 | FBLN1 |
| CYBB | CDCA3 | GPX4 | PLA2G6 | HELLS | ASAH2 |
| NOX3 | PEX2 | CISD1 | HIF1A | ENO1 | EMP1 |
| NOX4 | PEX6 | ATF4 | PARP1 | ECH1 | YY2 |
| NOX5 | TIMM9 | SCD | PARP2 | TIGAR | ESR1 |
| DUOX1 | DCAF7 | SRC | PARP3 | TXNRD1 | EP300 |
| DUOX2 | LCE2C | STAT3 | PARP4 | FDFT1 | ERCC6 |
| G6PD | FAR1 | PML | PARP6 | RELA | KDM5C |
| PGD | PHF21A | NFS1 | PARP8 | FSCN1 | UBE2D3 |
| VDAC2 | SMAD7 | TP63 | PARP9 | XRCC6 | ALAS2 |
| ACSL4 | LYRM1 | CDKN1A | PARP10 | CCT3 | DDB1 |
| LPCAT3 | AMN | FH | PARP11 | TNF | CCL5 |
| NRAS | PEX3 | CISD2 | PARP12 | SKP2 | RBX1 |
| KRAS | MTCH1 | SQSTM1 | PARP14 | ATG16L1 | CUL4A |
| HRAS | ACADSB | ISCU | PARP15 | ZFP36 | CUL4B |
| TP53 | GSK3B | FTH1 | PARP16 | H2AC1 | NFE2L1 |
| NCOA4 | MAPK8 | ACSL3 | PDSS2 | ADCY10 | NGLY1 |
| TF | SLC11A2 | CD44 | CREB1 | ERBB2 | SETDB1 |
| ALOX5 | PEBP1 | BRD4 | CREB3 | CTSB | H1-4 |
| ALOX12 | TGFB1 | PRDX6 | CREB5 | TLR4 | SEMA5A |
| ALOX12B | SNCA | CA9 | BEX1 | YWHAE | ABCB10 |
| ALOX15 | TFRC | LAMP2 | FABP4 | PKM | DCAF8 |
| ALOX15B | CGAS | PROM2 | AKT1S1 | NR1H4 | H1-5 |
| ALOXE3 | STING1 | CBS | MLST8 | RACK1 | H1-2 |
| PHKG2 | HDDC3 | GCH1 | CDH1 | RPL7 | H1-1 |
| EGFR | MDM2 | GCLC | SIRT1 | RPS3A | H1-3 |
| MAPK3 | MDM4 | RRM2 | SIRT6 | DPP4 | WDR76 |
| MAPK1 | POR | NR4A1 | KIF20A | CTH | COMMD10 |
| ZEB1 | DLD | PIK3CA | ETV4 | PRNP | H1-10 |
| SOCS1 | DNAJB6 | RPTOR | VCP | HMGCR | BACH1 |
| CDO1 | WWTR1 | SREBF1 | RBMS1 | GSS | ABCB6 |
| MYB | PRKCA | SREBF2 | KDM4A | SLC39A8 | LTF |
| HMOX1 | IFNG | P4HB | MGST1 | SLC39A14 | ADIPOR1 |
| SLC1A5 | SMPD1 | BCAT2 | MPC1 | ACSL5 | JAM3 |
| CHAC1 | MYCN | FXN | CAMKK2 | GCLM | H2AX |
| GOT1 | SMG9 | SUV39H1 | SRSF9 | SLC38A1 | YME1L1 |
| PRKAA2 | IL6 | ATF2 | MEF2C | ACSL6 | PLIN2 |
| PRKAA1 | PAQR3 | STK11 | NF2 | MAP1LC3A | RHOT1 |
| BAP1 | MICU1 | NEDD4L | HSPB1 | COQ2 | CSRP2 |
| ABCC1 | TOR2A | FTMT | EZH2 | SAT2 | STOML2 |
| ACVR1B | SIRT3 | BRD2 | PEDS1 | GJA1 | METTL14 |
| TGFBR1 | QSOX1 | BRD3 | CDC25A | PRKCB | DAZAP1 |
| TNFAIP3 | CLTRN | BRDT | CAV1 | HAMP | GABPB1 |
| ATF3 | KLF2 | DECR1 | LCN2 | PSEN1 | YTHDF2 |
| ATM | ELAVL1 | NCOA3 | TRIB2 | GGT1 | C19orf12 |
| IDH1 | YTHDC2 | NR5A2 | DHODH | SPARC | SLIRP |
| TAFAZZIN | DDR2 | MTOR | OTUB1 | MAT2A | ZSCAN25 |
| BECN1 | SLC39A7 | PANX2 | PDK4 | GPD2 | ALKBH5 |
| LONP1 | TRIM46 | TFAP2A | PTPN18 | FOXC1 | SCGB1D2 |
| CD82 | ACSL1 | CP | FTL | STYK1 | TMED8 |
| CYB5R1 | KDM5A | ARF6 | ABCC5 | NKAP | NOTCH2 |
| ELOVL5 | CYGB | GDF15 | CISD3 | PIR | TYRO3 |
| FADS1 | GSTZ1 | ABHD12 | GALNT14 | SESN2 | TRPV4 |
| FBXW7 | ACO1 | TFAM | KLHDC3 | VDAC1 | ATF6 |
| PTEN | SLC7A11 | KDM3B | GSTM1 | IDH2 | FZD7 |
| KEAP1 | CIRBP | RNF113A | RARRES2 | NFKB1 | BSG |
| AGPAT3 | YAP1 | PARK7 | USP11 | KAT5 | FXR1 |
| PEX12 | TRIM26 | AHCY | PTGS2 | DKK1 | SQLE |
| CHP1 | NDRG1 | PPARA | SLC3A2 | HNRNPA1 | TTBK2 |
| GPAT4 | FADS2 | SIAH2 | VDAC3 | JUN | ITGB8 |
| BRPF1 | LIFR | NEDD4 | PCBP1 | SNAI2 | LASP1 |
| OSBPL9 | PTPN6 | AIFM2 | MAP1LC3B | STUB1 | CEP290 |
| INTS2 | ADAM23 | PRDX1 | SAT1 | SOX2 | HLF |
| MMD | CPEB1 | AR | MAP1LC3C | GPX3 | PCDHB14 |
