# Supplementary material for: Establishment of the Diagnostic Signature of Ferroptosis Genes in Multiple Sclerosis
Source: Biochem Genet. 2024 Jun 17;63(4):3065–94. doi: 10.1007/s10528-024-10832-3 (PMC12271295; doi:10.1007/s10528-024-10832-3)
Supplement: Supplementary file 6 — Supplementary file6 (DOCX 15 KB) [file 10528_2024_10832_MOESM6_ESM.docx]

**Table S2. mRNA- miRNA interaction network nodes**

| mRNA |  | miRNA |
| --- | --- | --- |
| ATM | - | hsa-miR-4262 |
| ATM | - | hsa-miR-181c-5p |
| ATM | - | hsa-miR-181d-5p |
| ATM | - | hsa-miR-181a-5p |
| ATM | - | hsa-miR-181b-5p |
| GSK3B | - | hsa-miR-1297 |
| GSK3B | - | hsa-miR-214-3p |
| GSK3B | - | hsa-miR-3619-5p |
| GSK3B | - | hsa-miR-761 |
| GSK3B | - | hsa-miR-624-3p |
| GSK3B | - | hsa-miR-26a-5p |
| GSK3B | - | hsa-miR-26b-5p |
| GSK3B | - | hsa-miR-4465 |
| GSK3B | - | hsa-miR-3924 |
| GSK3B | - | hsa-miR-135b-5p |
| GSK3B | - | hsa-miR-135a-5p |
| HMGCR | - | hsa-miR-27a-3p |
| HMGCR | - | hsa-miR-513a-5p |
| HMGCR | - | hsa-miR-27b-3p |
| HMGCR | - | hsa-miR-493-5p |
| KLF2 | - | hsa-miR-101-3p |
| MAPK1 | - | hsa-miR-520d-5p |
| MAPK1 | - | hsa-miR-524-5p |
| MAPK1 | - | hsa-miR-543 |
| MAPK1 | - | hsa-miR-3619-5p |
| MAPK1 | - | hsa-miR-212-3p |
| MAPK1 | - | hsa-miR-132-3p |
| MAPK1 | - | hsa-miR-541-5p |
| MAPK1 | - | hsa-miR-214-3p |
| MAPK1 | - | hsa-miR-301a-3p |
| MAPK1 | - | hsa-miR-301b-3p |
| MAPK1 | - | hsa-miR-3666 |
| MAPK1 | - | hsa-miR-4295 |
| MAPK1 | - | hsa-miR-130a-3p |
| MAPK1 | - | hsa-miR-761 |
| MAPK1 | - | hsa-miR-130b-3p |
| MAPK1 | - | hsa-miR-454-3p |
| MAPK1 | - | hsa-miR-5691 |
| MAPK1 | - | hsa-miR-106b-5p |
| MAPK1 | - | hsa-miR-488-3p |
| NFE2L1 | - | hsa-miR-4524a-5p |
| NFE2L1 | - | hsa-miR-4524b-5p |
| NRAS | - | hsa-miR-4500 |
| NRAS | - | hsa-miR-4458 |
| NRAS | - | hsa-let-7f-5p |
| NRAS | - | hsa-let-7d-5p |
| NRAS | - | hsa-miR-98-5p |
| NRAS | - | hsa-let-7b-5p |
| NRAS | - | hsa-let-7g-5p |
| NRAS | - | hsa-let-7a-5p |
| NRAS | - | hsa-let-7i-5p |
| NRAS | - | hsa-let-7e-5p |
| NRAS | - | hsa-let-7c-5p |
| NRAS | - | hsa-miR-2278 |
| NRAS | - | hsa-miR-425-5p |
| NRAS | - | hsa-miR-514a-5p |
| PIK3CA | - | hsa-miR-186-5p |
| PIK3CA | - | hsa-miR-19a-3p |
| PIK3CA | - | hsa-miR-522-3p |
| PIK3CA | - | hsa-miR-224-3p |
| PIK3CA | - | hsa-miR-19b-3p |
| PIK3CA | - | hsa-miR-200c-3p |
| PIK3CA | - | hsa-miR-429 |
| PIK3CA | - | hsa-miR-200b-3p |
| PIK3CA | - | hsa-miR-3121-3p |
| PIK3CA | - | hsa-miR-664b-3p |
| PIK3CA | - | hsa-miR-579-3p |
| PIK3CA | - | hsa-miR-320d |
| PIK3CA | - | hsa-miR-4429 |
| PIK3CA | - | hsa-miR-320c |
| PIK3CA | - | hsa-miR-320b |
| VDAC3 | - | hsa-miR-1286 |
| VDAC3 | - | hsa-miR-629-5p |
| VDAC3 | - | hsa-miR-9-5p |
| VDAC3 | - | hsa-miR-7-5p |

“mRNA” and “miRNA” represent node；

“-” represent edge
