# Supplementary material for: Establishment of the Diagnostic Signature of Ferroptosis Genes in Multiple Sclerosis
Source: Biochem Genet. 2024 Jun 17;63(4):3065–94. doi: 10.1007/s10528-024-10832-3 (PMC12271295; doi:10.1007/s10528-024-10832-3)
Supplement: Supplementary file 7 — Supplementary file7 (DOCX 12 KB) [file 10528_2024_10832_MOESM7_ESM.docx]

**Table S3. mRNA-TF interaction network nodes**

| mRNA |  | TF |
| --- | --- | --- |
| ATM | - | RUNX3 |
| GSK3B | - | HDAC1 |
| HMGCR | - | TCF3 |
| KLF2 | - | STAT5B |
| MAPK1 | - | IRF4 |
| MAPK1 | - | MEF2A |
| MAPK1 | - | PBX3 |
| MAPK1 | - | POU2F2 |
| MAPK1 | - | TBL1XR1 |
| MAPK1 | - | TCF12 |
| MAPK1 | - | BCL11A |
| NFE2L1 | - | BCL11A |
| NFE2L1 | - | LMO2 |
| NFE2L1 | - | PAX5 |
| NFE2L1 | - | TCF3 |
| PCBP1 | - | RUNX1T1 |
| PCBP1 | - | RUNX3 |
| PCBP1 | - | CDK7 |
| PCBP1 | - | IRF4 |
| PCBP1 | - | LMO2 |
| PCBP1 | - | MYB |
| PIK3CA | - | BCL11A |
| PIK3CA | - | RUNX3 |
| RPL8 | - | MYB |
| RPL8 | - | RUNX3 |
| RPL8 | - | TCF3 |
| RPL8 | - | TFAP4 |
| VDAC3 | - | RUNX3 |
| VDAC3 | - | TCF3 |
| VDAC3 | - | ZEB1 |
| VDAC3 | - | ZNF384 |

“mRNA” and “TF” represent node；

“-” represent edge；

TF：Transcription factors
